# Post-exposure treatment with whole inactivated H5N1 avian influenza virus protects against lethal homologous virus infection in mice

**DOI:** 10.1038/srep29433

**Published:** 2016-07-12

**Authors:** Mable Hagan, Charlene Ranadheera, Jonathan Audet, Jocelyn Morin, Anders Leung, Darwyn Kobasa

**Affiliations:** 1 Department of Medical Microbiology, University of Manitoba, Winnipeg, Manitoba, Canada; 2 High Containment Respiratory Viruses, Special Pathogens Program, Public Health Agency of Canada, Winnipeg, Manitoba, Canada; 3 Département des Technologies de laboratoire, Cégep de Sherbrooke, Sherbrooke, Québec, Canada

## Abstract

Concerns with H5N1 influenza viruses include their prevalence in wild and domestic poultry, high mortality rate (~60%) in humans with some strains, lack of pre-existing immunity in humans, and the possibility that these viruses acquire mutations that enable efficient transmission between humans. H5 subtype viruses of Eurasian origin have recently appeared in wild and domestic bird populations in North America, and have led to the generation of new virus strains that are highly pathogenic in poultry. These new H5 HA containing viruses with their ability to evolve rapidly represent an unknown threat to humans in contact with infected poultry, and vaccination with an off-the-shelf vaccine may be impractical to provide protection to at-risk individuals. Instead, we have evaluated the efficacy of a formalin-inactivated vaccine, which could be derived directly from a circulating virus, to provide post-exposure protection. This strategy was evaluated using a prototypic highly pathogenic avian H5N1 strain, A/Vietnam/1203/2004, and demonstrated rapid induction of adaptive immune responses providing protection in a mammalian model of lethal infection. Additionally, this post-exposure vaccine was highly efficacious when administered 24 hours after exposure. This study offers a platform for developing effective post-exposure vaccines for treatment of highly virulent influenza infections.

Highly pathogenic avian influenza (HPAI) H5N1 viruses are found in several countries in Asia, Europe, Africa, the Middle East, and more recently, North America^1^. Transmission to humans occurs through contact with infected birds, and as of December 2015, there have been 844 confirmed human cases of H5N1 virus infection and 449 deaths (53% case fatality rate^2^). In early 2014, a novel HPAI H5N8 virus began circulating globally in poultry populations^3^ and by late 2014, this virus reassorted with a North American avian virus into a novel HPAI H5N2 that caused the first North American outbreak of a Eurasian lineage HPAI virus in poultry^4^. The presence of these novel viruses in North American poultry flocks for the first time presents a new risk for exposure, and potentially human disease. Although human-to-human transmission of H5N1 viruses is rare, if it has occurred at all, the naturally high mutation rates of these viruses coupled with their ability to reassort gene segments^5^ may lead to adaptation to human host receptors and the generation of new pandemic viruses.

Predicting the strain of H5 virus which could emerge as a new pandemic virus is challenging. Although H5 strains are highly variable, the WHO has recommended the production and stockpiling of H5N1 vaccines based on existing strains as part of their global influenza pandemic preparedness plan^6^. However, to vaccinate individuals at high risk for H5N1 virus infection in advance against a strain that has not yet emerged is unrealistic. As a result, the first effective response to an epidemic or pandemic is to use antivirals to help control the spread of disease. However, current antivirals (oseltamivir and zanamivir) must be administered early after infection for optimal efficacy and are highly susceptible to development of resistance^7^,^8^,^9^. Thus, to be better prepared for future H5 pandemics, it is advantageous to develop new post-exposure strategies that could be used therapeutically.

Studies have shown that inactivated whole H5N1 virus given as a pre-exposure vaccine can protect mice from homologous and heterologous challenge with HPAI H5N1 viruses^10^,^11^,^12^, and can protect against challenge in other animal models including ferrets^13^,^14^,^15^ and non-human primates^16^. In contrast, post-exposure vaccines against HPAI H5 viruses have not been investigated, even though post-exposure vaccines against other viruses such as rabies, hepatitis B, and smallpox have worked successfully^17^,^18^,^19^. The strategy of using vaccines to provide post-exposure prophylaxis and therapy is increasingly viewed as a viable option for outbreak response. Efficacy of post exposure vaccination has been demonstrated, even against highly acute viral infections such as that caused by Ebola virus^20^,^21^, showing promise to effectively interfere with disease progression when used early after infection. To date, there has been one successful post-exposure study using live recombinant vesicular stomatitis virus expressing hemagglutinin (HA) from A/Puerto Rico/8/34 (H1N1; PR8) strain, a lab-adapted strain with high growth properties^22^,^23^,^24^, to provide complete protection in mice infected with a lethal dose of PR8 up to 24 hours post-exposure^25^.

As a proof-of-concept study, we wanted to evaluate the efficacy of using whole inactivated H5N1 vaccine as a post-exposure treatment against lethal H5N1 infection. Using a formalin inactivated A/Vietnam/1203/2004 (H5N1, clade 1) (V1203) virus, we examined the ability of whole inactivated V1203 (WI-V1203) to provide post-exposure protection in mice from lethal homologous infection, the minimal dose required to provide 100% protection, the role of cell mediated or humoral immunity in the protective response, and how long after infection can WI-V1203 be administered and still provide protection. We showed that post-exposure treatment with WI-V1203 resulted in faster clearance of viral infection, rapid production of neutralizing antibodies, and complete protection when provided immediately after infection. Furthermore, protection could still be achieved when administered up to 1 day post-infection, offering a new strategy to protect high-risk individuals in an outbreak. These results also propose the potential utility of using pre-existing safety approved H5N1 vaccines for the purpose of post-exposure prophylaxis, extending the use of already stockpiled vaccines in the event of a pandemic.

## Results

### Post-exposure treatment with WI-V1203 protects against homologous infection and is dose-dependent

**Figure 1:**
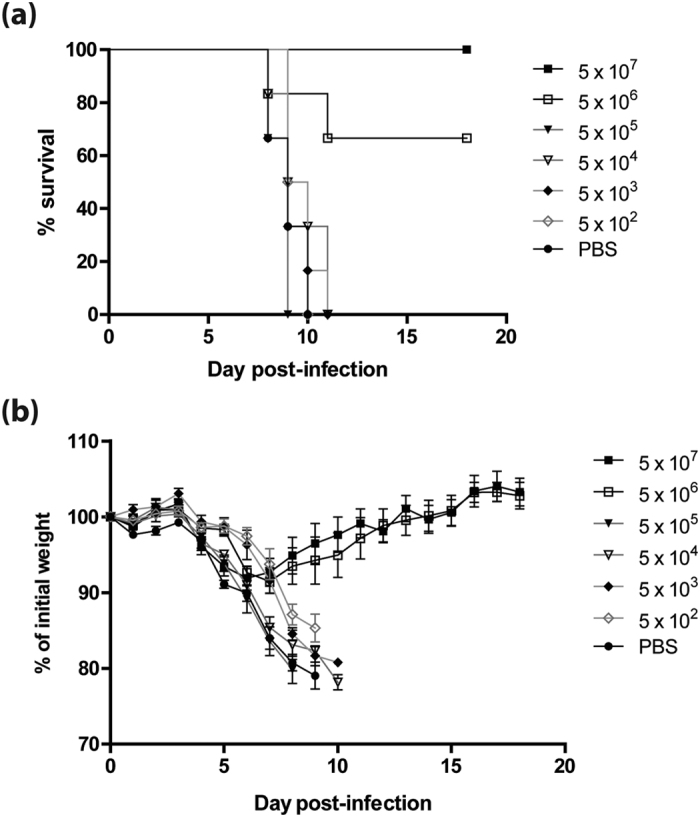
Protective efficacy of post-exposure treatment with WI-V1203 against lethal homologous H5N1 infection. Mice were infected IN with 3LD_50_ (3 PFU) of A/Vietnam/1203/2004 (H5N1) and treated with either an IM dose of WI-V1203 that ranged from 5 × 10^2^ to 5 × 10^7^ PFUeq or PBS. (**a**) Survival and (**b**) weight loss was measured for 18 days post-infection. Each data point is the average from 6 mice ± SEM.

To evaluate the ability of WI-V1203 to protect mice from H5N1 infection, groups of mice (n = 6) were first infected via the intranasal (IN) route with a lethal dose of V1203 (3 LD_50_) and immediately treated with an intramuscular (IM) injection of WI-V1203 or PBS. A dose range of WI-V1203 vaccine equivalent to the virus contained in 5 × 10^2^ to 5 × 10^7^ PFUs of non-inactivated V1203 virus (expressed as PFUeq) was tested to determine the minimum dose required to offer full protection. All of the mice treated with the highest dose (5 × 10^7^ PFUeq) of WI-V1203 survived (Fig. 1a), showing no signs of disease and minimal weight loss (Fig. 1b). At the second highest dose (5 × 10^6^ PFUeq) 4 of 6 mice survived with minimal weight loss. For all lower doses, greater weight loss of up to 25% and signs of CNS disease, typical of H5N1 infection in mice, was observed. Mice either died from infection or were euthanized due to severity of disease signs by day 11, similar to PBS-treated mice. From these results, a dose-dependent protective response was observed, and a high dose of 5 × 10^7^ PFUeq of WI-V1203 was necessary to provide 100% protection in mice.

### Treatment with WI-V1203 reduces virus spread and replication

To examine the protective response elicited by WI-V1203 treatment against lethal H5N1 infection, a serial sacrifice study was performed. Mice were infected with 3 LD_50_ of V1203 and immediately treated with an IM injection of 5 × 10^7^ PFUeq of WI-V1203 [(+)infected/(+)treated] or PBS [(+)infected/(−)treated]. Groups of mice (n = 6) were serially sacrificed at 2, 4, 6, 8, and 10 days post-infection (dpi) to measure virus replication in lungs, virus dissemination to the spleen as a known extra pulmonary target of H5N1 virus, antibody levels, and T cell responses.

**Figure 2:**
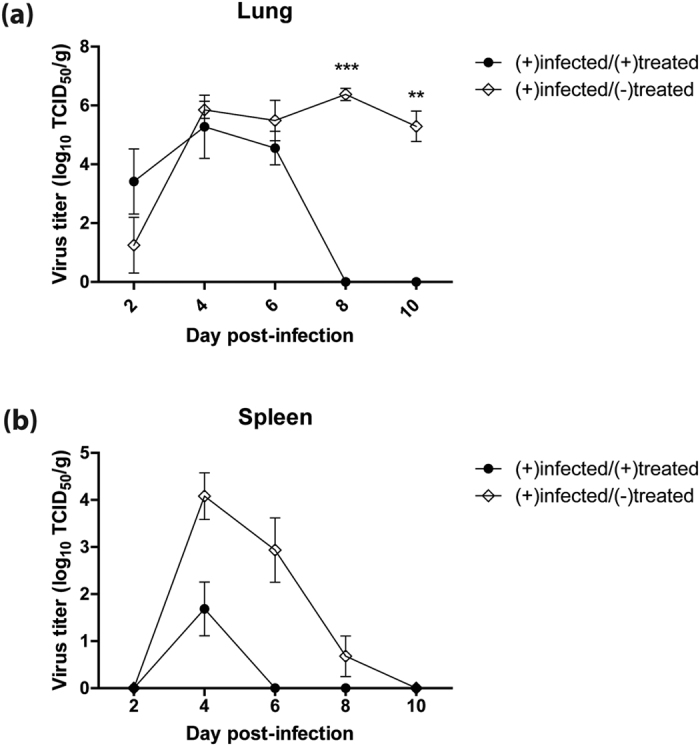
Effect of WI-V1203 post-exposure treatment on H5N1 replication in mice. Mice were infected IN with 3LD_50_ (3 PFU) of A/Vietnam/1203/2004 (H5N1) and treated with either 5 × 10^7^ PFUeq of WI-V1203 [(+)infected/(+)treated] or PBS [(+)infected/(−)treated] by IM injection. Mice were sacrificed at each time point, and virus titers in the (**a**) lung and (**b**) spleen were determined by TCID_50_ assay. Each data point represents the average of 6 mice ± SEM. 2-way ANOVA analysis with Bonferroni post-tests were performed and statistical significance was determined and shown **p-value < 0.01, ***p-value < 0.001.

At 2 dpi, although the (+)infected/(+)treated group had a lung titer of 10^3.4^ TCID_50_/g and the (+)infected/(−)treated group had a titer of 10^1.3^ TCID_50_/g, these values were not significantly different (Fig. 2a, 2-way ANOVA with Bonferroni post-tests, p-value > 0.05). Major differences were observed on 8 and 10 dpi, where the (+)infected/(−) treated group continued to show significantly high levels of virus in the lungs in contrast to the (+)infected/(+)treated group that cleared the virus by 8 dpi (p-value < 0.001 at 8 dpi, p-value < 0.01 at 10 dpi). In the spleen at 4 dpi, the (+)infected/(+)treated group had a virus titer that was ~250-fold lower compared to the (+)infected/(−)treated group (Fig. 2b). By 6 dpi, while virus was still present in the spleen for the (+)infected/(−)treated group, no virus was detected in the (+)infected/(+)treated group. Overall, WI-V1203 treated mice were able to rapidly clear the H5N1 infection, showing increased survival and recovery to their initial weights.

### Reduced activation of CD4+ and CD8+ T cells in WI-V1203 treated mice

**Figure 3:**
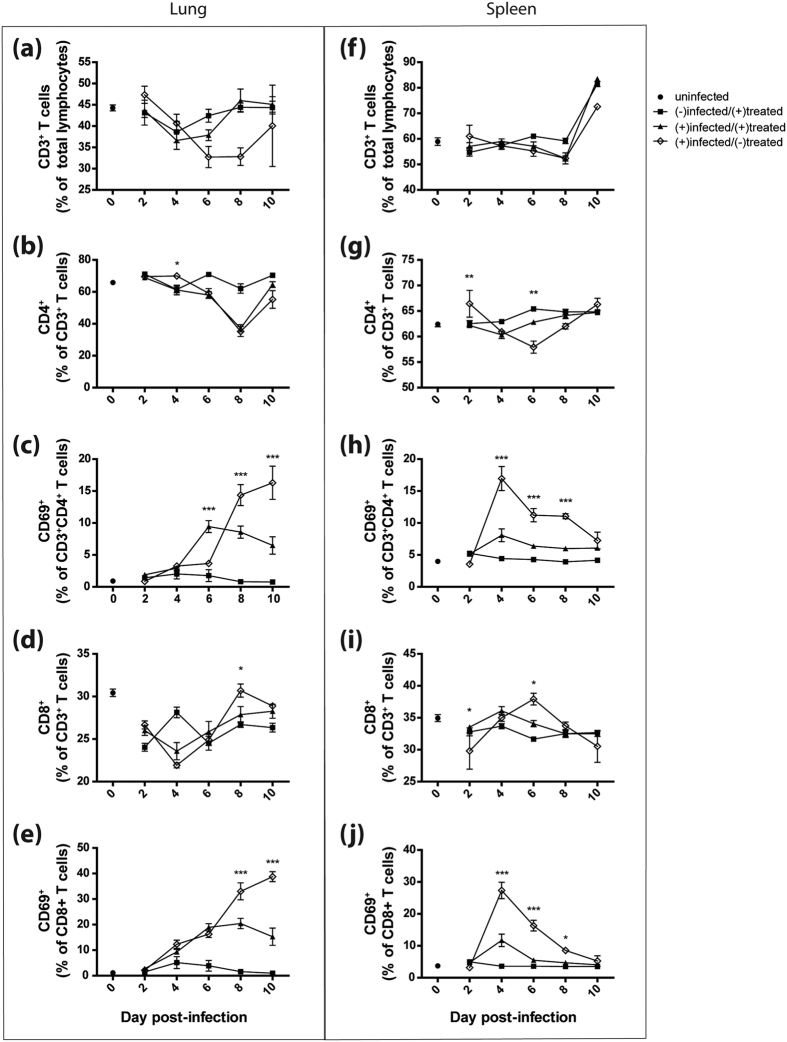
CD4 and CD8 T cell responses in mice infected with H5N1. Mice were infected IN with 3LD_50_ (3 PFU) of V1203 and treated with either WI-V1203 [(+)infected/(+)treated] or PBS [(+)infected/(−)treated] by IM injection. Uninfected and untreated mice were sacrificed on day 0 to determine baseline levels. As treatment controls, a group of mice were mock-infected with virus diluent and treated with WI-V1203 [(−)infected/(+)treated]. Mice were sacrificed at each time point and lungs (**a**–**e**) and spleens (**f**–**j**) were analyzed by flow cytometry to measure CD3+ (**a**,**f**), CD3+CD4+ (**b**,**g**), and CD3+CD8+ (**c**, **h**) expressing T cells. T cell activation was measured by the detection of CD69 (**c**,**e**,**h**,**j**). Each data point represents the average of 6 mice ± SEM. 2-way ANOVA analysis with Bonferroni post-tests were performed and statistical significance between (+)infected/(+)treated and (+)infected/(−)treated are shown *p-value < 0.05, **p-value < 0.01, ***p-value < 0.001.

The contribution of T cells to the protective response observed in mice treated with WI-V1203 were measured by flow cytometry on 2, 4, 6, 8, and 10 dpi (n = 6/day/group). Three groups of mice were examined: virus diluent mock-infected and WI-V1203 treated [(−)infected/(+)treated], V1203 infected and WI-V1203 treated [(+)infected/(+)treated], and V1203 infected and PBS mock-treated [(+)infected/(−)treated]. To establish baseline levels of T cell responses, a group of uninfected and untreated mice (n = 4) were sacrificed on day 0. The percentage of T cells, indicated by CD3 staining, from the total lymphocyte population in the lungs of each group of mice was determined (Fig. 3a). For both the (−)infected/(+)treated and (+)infected/(+)treated groups, the overall number of CD3+ T cells in the lungs were initially similar compared to the (+)infected/(−)treated mice, although this latter group had fewer total T cells as the days progressed. In the lungs, CD4+ T cells in the (−)infected/(+)treated group remained close to the baseline level throughout the 10 days, while both infected groups regardless of treatment showed a drop in CD4+ T cells from ~60% at 6 dpi to ~35% at 8 dpi, that rebounded by 10 dpi (Fig. 3b).

The surface marker CD69, which identifies activated T cells that produce T-helper-1 (Th1)-like cytokines such as IL-2, TNF-α, and IFN-γ^26^,^27^, was used to determine the percentage of T cell activation. In the lungs, there was minimal CD4+ T cell activation in the (−)infected/(+)treated group at all time points (Fig. 3c). Major differences in CD4+ T cell activation in the other groups appeared on 6 dpi, with significantly higher levels of CD4+ CD69+ T cells in the (+)infected/(+)treated group compared to the (+)infected/(−)treated group (2-way ANOVA with Bonferroni post-tests, p-value < 0.001). Interestingly, activated T cells in the (+)infected/(+)treated group remained constant until 8 dpi and then declined by 10 dpi. This was opposite to the (+)infected/(−)treated group where the level of CD4+ T cell activation increased significantly on 8 and 10 dpi (Fig. 3c, p-value < 0.001 for 8 and 10 dpi).

The percentage of CD8+ T cells in the lungs decreased for all groups in comparison to the baseline value (Fig. 3d). The only difference between the infected groups was observed on 8 dpi, where the (+)infected/(+)treated group had significantly lower CD8+ T cells compared to the (+)infected/(−)treated group (2-way ANOVA with Bonferroni post-tests, p-value < 0.05). Like the lung CD4+ T cell activation data (Fig. 3c), CD8+ T cell activation was also significantly higher in the (+)infected/(−)treated group on 8 and 10 dpi compared to the (+)infected/(+)treated group (Fig. 3e, p-value < 0.001 for 8 and 10 dpi).

In the spleen, CD3+ T cell levels were determined from the total lymphocyte population and no differences were observed between all groups (Fig. 3f). The percentage of CD4+ T cells remained at similar levels for both WI-V1203 treated groups between 2 and 10 dpi regardless of whether the animals were infected, and were similar to the baseline control (Fig. 3g). However, the (+)infected/(−)treated group exhibited a decrease in CD4+ T cells between 2 and 6 dpi followed by an increased level between 6 and 10 dpi. As in the lung, little activation of CD4+ T cells was observed in the (−)infected/(+)treated group, only modest activation in the (+)infected/(+)treated group, but, substantial activation occurred in the (+)infected/(−)treated group (Fig. 3h). The increases in the latter group occurred earlier in the spleen peaking at 4 dpi and then decreasing, as opposed to the increasing level of activated CD4+ T cells in the lung seen at each day after 4 dpi (Fig. 3c).

Minor differences were seen in CD8+ T cell levels at all time points for the WI-V1203-treated groups (Fig. 3i). However, the abundance of CD8+ T cells in the (+)infected/(−)treated group followed a pattern opposite to the CD4+ T cells, starting at lower levels than the other groups on 2 dpi, increasing above the other groups by 6 dpi and then dropping again by 10 dpi (Fig. 3i). For all groups the percentage of activated CD8+ T cells (Fig. 3j) mirrored that seen for activated CD4+ T cells. Overall, WI-V1203 treatment without infection showed no activation of T cells in the lung or spleen. Activation of T cells as a consequence of infection was seen for both infected groups, but the (+)infected/(+)treated group showed less activation of T cells despite clear protection from infection, while the (+)infected/(−)treated group had substantially higher T cell activation at later time points. The complete data set on total cell counts for each sample is provided as Supplementary Data to illustrate the size of T cell populations found in each sample, although our procedure did not permit the measurement of the magnitude of the total T cell response.

### Enhanced antibody responses in mice infected with H5N1 and treated with WI-V1203

**Figure 4:**
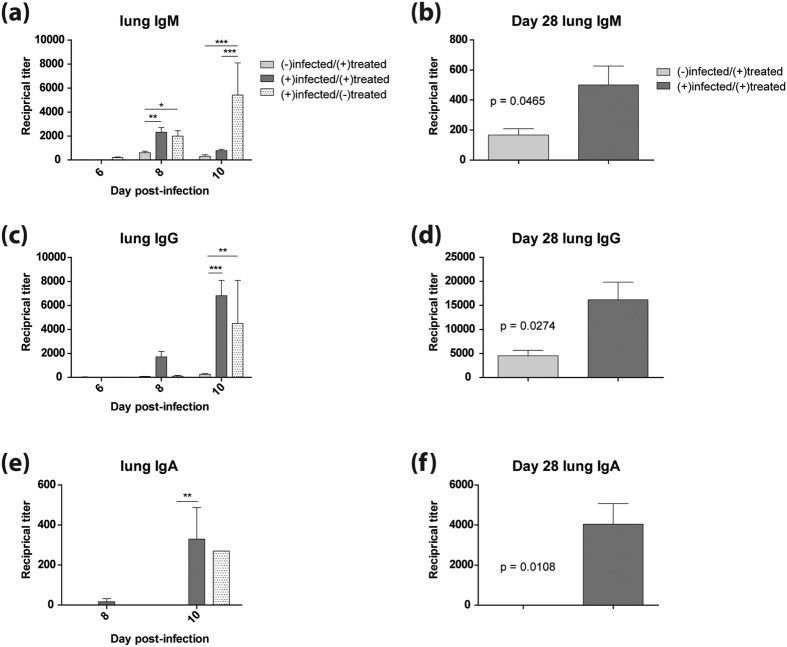
Lung antibody responses in mice infected with H5N1. Mice were infected IN with 3LD_50_ (3 PFU) of V1203 and treated with either WI-V1203 [(+)infected/(+)treated] or PBS [(+)infected/(−)treated] by IM injection. Uninfected and untreated mice (n = 4) were sacrificed on day 0 to determine baseline levels. A group of mice were mock-infected with MEM + 0.1%BSA and treated with WI-V1203 as treatment controls [(−)infected/(+)treated]. Mice (n = 6) were sacrificed at each time point and levels of IgM (**a**,**b**), IgG (**c**,**d**), and IgA (**e**,**f**) in lung homogenates were determined by ELISA. 2-way ANOVA analysis with Bonferroni post-tests (**a**,**c**,**e**) and 2-sided unpaired t-tests with Welch’s correction (**b**,**d**,**f**) were performed and statistical significance are shown as *p-value < 0.05, **p-value < 0.01, ***p-value < 0.001.

To determine the humoral immune response involvement in providing protection to WI-V1203 treated mice, V1203-specific IgM, IgG, and IgA in the lungs and serum were measured by ELISA. At 8 dpi in the lungs, a minimal IgM response was detected for the (−)infected/(+)treated mice compared to significantly higher IgM titers found in the (+)infected/(+)treated group with a reciprocal titer of 2300 (p-value < 0.01), and the (+)infected/(−)treated group with a titer of 2000 (p-value < 0.05) (Fig. 4a, 2-way ANOVA with Bonferroni post-tests). Interestingly, by 10 dpi, the IgM response for the (+)infected/(+)treated group receded while the (+)infected/(−)treated group increased significantly (p-value < 0.001). At 28 dpi, the (+)infected/(+)treated group had a significantly higher IgM response compared to the (−)infected/(+)treated group (Fig. 4b, t-test, t = 2.500, df = 6, p-value = 0.0465). For the lung IgG responses, at 8 dpi only the (+)infected/(+)treated group had any detectable titers (Fig. 4c). By 10 dpi, the (+)infected/(+)treated group had significantly higher IgG titers (2-way ANOVA with Bonferroni post-tests, p-value < 0.001) along with the (+)infected/(−)treated group (p-value < 0.01,) compared to the (−)infected/(+)treated group. Like the IgM response at 28 dpi, the IgG response for the (+)infected/(+)treated group was significantly higher than the (−)infected/(+)treated group (Fig. 4d, t-test, t = 3.081, df = 5, p-value = 0.0274). This data demonstrates that IgG responses developed prominently in response to infection, and vaccine treatment further promoted rapid maturation of antibody responses from IgM to IgG.

The lung IgA response followed the same trend as IgG, where the (+)infected/(+)treated group had the earliest detectable titer, at 8 dpi, that increased to significantly higher IgA titers compared to the (−)infected/(+)treated group by 10 dpi (Fig. 4e, 2-way ANOVA with Bonferroni post-tests, p-value < 0.01). The (+)infected/(−)treated group, however, had detectable IgA titers only at 10 dpi. Similar to IgM and IgG, at 28 dpi the (+)infected/(+)treated group had significantly higher IgA response compared to the (−)infected/(+)treated group (Fig. 4f, t-test, t = 3.953, df = 5, p-value = 0.0108).

**Figure 5:**
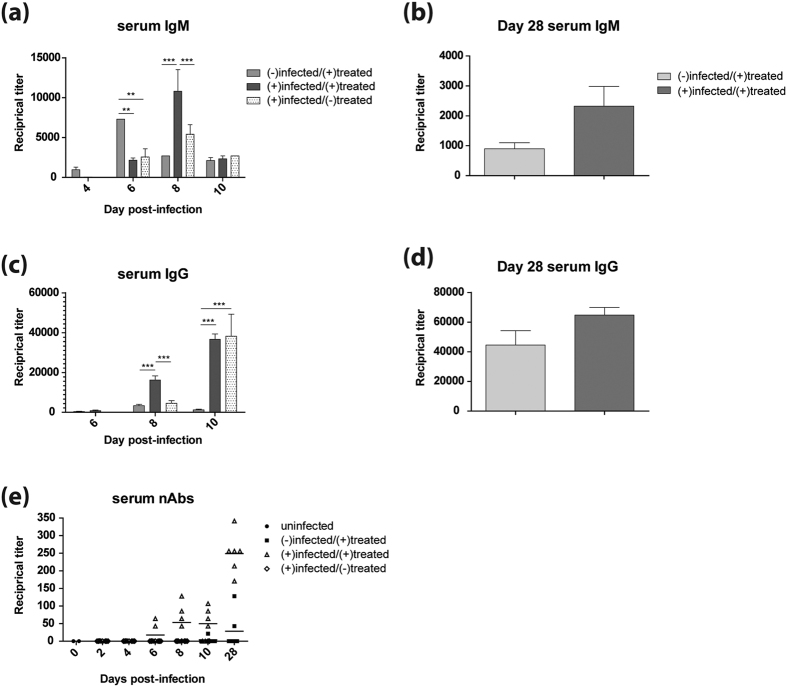
Serum antibody responses in mice infected with H5N1. Mice were infected IN with 3LD_50_ (3 PFU) of V1203 and treated with either WI-V1203 [(+)infected/(+)treated] or PBS [(+)infected/(−)treated] by IM injection. Uninfected and untreated mice (n = 4) were sacrificed on day 0 to determine baseline levels. A group of mice were mock-infected with MEM + 0.1%BSA and treated with WI-V1203 as treatment controls [(−)infected/(+)treated]. Mice (n = 6) were sacrificed at each time point and levels of IgM (**a**,**b**) and IgG (**c**,**d**) in the serum were determined by ELISA. Serum neutralizing antibody titers (**e**) were determined by microneutralization assay. 2-way ANOVA analysis with Bonferroni post-tests (**a**,**c**) and 2-sided unpaired t-tests with Welch’s correction (**b**,**d**) were performed and statistical significance are shown as **p-value < 0.01 or ***p-value < 0.001.

Levels of serum IgM and IgG from V1203 infection with WI-V1203 treatment were similar to the response seen in the lungs. Although minimal levels of IgM in the (−)infected/(+)treated group were detected in the lungs (Fig. 4a), serum titers were significantly higher, peaking at 6 dpi (Fig. 5a, 2-way ANOVA with Bonferroni post-tests, p-value < 0.01). For both the infected groups, , IgM titers peaked at 8 dpi and were significantly higher for the (+)infected/(+)treated animals than the (−)infected/(+)treated and (+)infected/(−)treated groups (p-value < 0.001). On 28 dpi, IgM titers for (−)infected/(+)treated and (+)infected/(+)treated were not significantly different (Fig. 5b, t-test, t = 2.061, df = 5, p-value = 0.0943). Similar to lung IgG levels, serum IgG for the (+)infected/(+)treated group was significantly greater than (−)infected/(+)treated and (+)infected/(−)treated groups on 8 dpi (Fig. 5c, 2-way ANOVA with Bonferroni post-tests, p value < 0.001). Serum IgG titers further increased by 10 dpi to similar levels for (+)infected/(+)treated and (+)infected/(−)treated groups, which were both significantly greater than the (−)infected/(+)treated group (p-value < 0.001). At 28 dpi, serum IgG titers were also not significantly different between (−)infected/(+)treated and (+)infected/(+)treated groups (Fig. 5d, t-test, t = 1.838, df = 7, p-value = 0.1087).

Microneutralization assays were used to determine the levels of neutralizing antibody (nAb) activity associated with serum antibody levels. Only mice from the (+)infected/(+)treated group had nAbs that appeared as early as 6 dpi, with titers in two of the six mice, and by 8 and 10 dpi, four of the six mice on each day had nAb titers that ranged from 42.7 to 106.7 (Fig. 5e). Sera from the (+)infected/(+)treated group on 28 dpi all showed high levels of nAbs, with an average titer of 256. These results show the relationship between survival of the (+)infected/(+)treated group of mice and the production of nAbs. Strikingly, the (−)infected/(+)treated group had only one mouse with a titer of 21.3 at 10 dpi, and by 28 dpi, only two of the six mice had nAbs with an average titer of 28.4 (Fig. 5e). These data show the enhancement provided by infection and vaccine-treatment, which stimulated faster production of total IgG and IgM antibodies with greater neutralizing capabilities, over vaccination alone.

### WI-V1203 vaccine treatment provides protection up to 1 day post-infection

**Figure 6:**
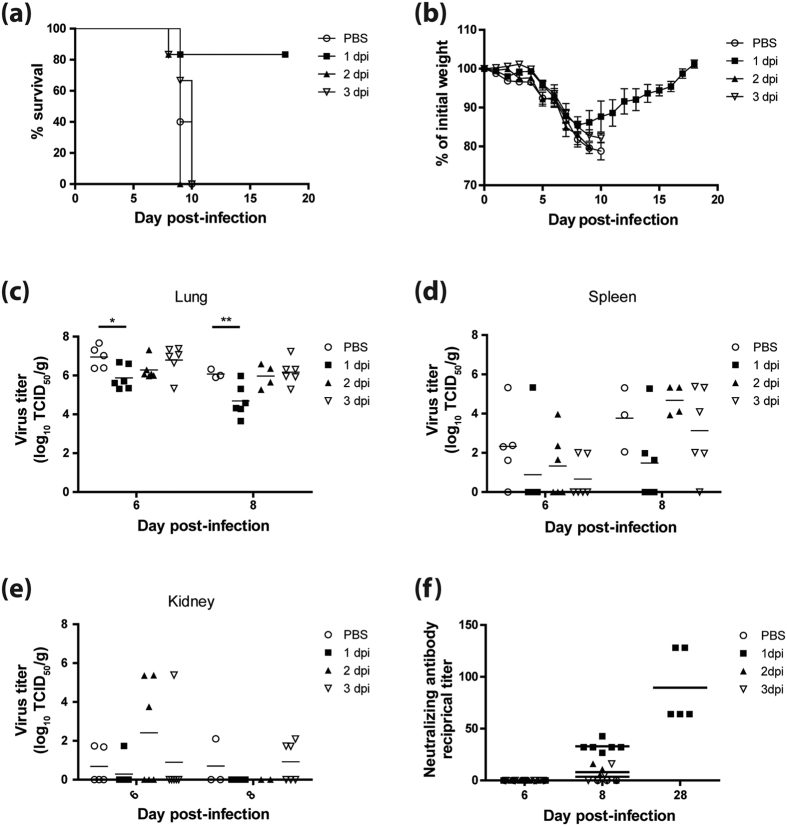
Effect of WI-V1203 treatment administered 1, 2, or 3 days post-exposure on survival of mice infected with lethal H5N1. Mice were infected IN with 5LD_50_ of A/Vietnam/1203/2004 (H5N1) and treated IM with 5 × 10^7^ PFUeq of WI-V1203 at 1, 2, or 3 dpi. A group of mice (n = 5) were treated with PBS at 1 dpi as an infection control. (**a**) Survival and (**b**) weight loss of the treated groups were monitored for 18 days post-infection. Mice (n = 6/day) were sacrificed at days 6 and 8 post-infection to determine viral replication in the (**c**) lung, (**d**) spleen and (**e**) kidney by TCID_50_ assay. (**f**) Neutralizing antibodies were also detected in the serum by microneutralization assay. 2-way ANOVA analysis with Bonferroni post-tests (**c–f**) were performed and statistical significance was determined and shown as *p-value < 0.05, **p-value < 0.01.

To assess the time frame in which WI-V1203 treatment can provide protection, mice were infected with a lethal dose of V1203 and treated with WI-V1203 at 1, 2, and 3 dpi (n = 6 per group/day) or mock-treated at 1 dpi (n = 5 per day). Five of six mice treated with WI-V1203 at 1 dpi survived with 16% maximum weight loss and full recovery to their initial weight by 18 dpi. Mice that were treated 2 and 3 dpi did not survive, and showed similar disease progression and weight loss as the mock-treated controls (Fig. 6a,b).

H5N1 HPAI viruses typically cause systemic infection in mice, therefore, mice from each group were sacrificed 6 and 8 dpi to collect spleen, kidney, and lung tissues for virus titration. At 6 dpi, lungs of the mock-treated mice had the greatest virus titers, followed by the 3 dpi and 2 dpi WI-V1203-treated groups (Fig. 6c). Surprisingly, mice treated 1 dpi with WI-V1203 also had considerable virus in their lungs at 6 dpi; however, these values were significantly lower than virus titers in the mock-treated mice (2-way ANOVA with Bonferroni post-tests, p-value < 0.05). By 8 dpi, the 1 dpi WI-V1203-treated group showed further significant reduction in lung titers in comparison to the mock-treated group (p-value < 0.01), while titers remained similar for the 2 and 3 dpi WI-V1203-treated groups.

In the spleen, 80% of the mock-treated mice had virus titers at 6 dpi (Fig. 6d). For the 1 dpi WI-V1203-treated group, virus was detected in 16.7% of mice compared to 50% and 33% for the 2 and 3 dpi WI-V1203 treated mice, respectively. By 8 dpi, two mock-treated mice died, and the three remaining all had virus in the spleen. The 1 dpi WI-V1203-treated group had the lowest spleen titers, present in only 50% of the mice. The 2 dpi WI-V1203-treated group had 4 survivors, all with virus in the spleen, while five of the six 3 dpi WI-V1203-treated group also had virus detected in the spleen (Fig. 6d).

Virus dissemination to the kidneys was much lower in all groups than for the spleen. At 6 dpi, 40% of mock-treated mice had low viral titers in their kidneys (Fig. 6e). One mouse in each of the 1 and 3 dpi WI-V1203-treatment groups and 3 mice in the 2 dpi WI-V1203-treatment group also had kidney viral titers at 6 dpi. By 8 dpi, no virus was detected in the 1 dpi WI-V1203 treated group. Despite the higher frequency of virus detected in the 2 dpi WI-V1203-treated group at 6 dpi, none of the surviving mice in this group had virus detected by 8 dpi. This is in contrast to the 3 dpi WI-V1203-treated group where 3 of the 6 mice continued to show low titers.

To examine the contribution of nAb in humoral-mediated protection, nAbs were measured in the serum of infected mice. At 6 dpi, none of the groups had any level of nAbs (Fig. 6f). Interestingly, at 8 dpi all of the mice treated with WI-V1203 at 1 dpi had low levels of nAbs. For the 2 dpi WI-V1203-treated group, three out of four remaining mice also had detectable, but lower, nAbs titers, while the 3 dpi WI-V1203-treated group had only two mice with nAb titers. The 1 dpi WI-V1203-treatment group was the only group with survivors at 28 dpi, and all five mice had an average nAb titer of 89.6 (Fig. 6f). This response was noticeably less than the nAb titer of 248.9 in mice that received WI-V1203 treatment immediately after exposure to lethal V1203 infection (Fig. 5e).

Overall, 83.3% mice treated with WI-V1203 at 1 dpi survived lethal V1203 infection, while treatment with WI-V1203 at 2 or 3 dpi was unable to provide protection (Fig. 6a). Corresponding with increased survival, the mice treated at 1 dpi with WI-V1203 had reduced viral titers in the lung, spleen and kidney compared to the mock-treated control group and mice from the other two treatment groups (Fig. 6c–e). Furthermore, all mice from the 1 dpi WI-V1203-treated group had low levels of serum nAbs at 8 dpi compared to the mock-treated group which had no nAb response (Fig. 6f). Although the 2 and 3 dpi treatment groups had a few animals with nAbs in the serum at 8 dpi, these lower levels of nAbs proved to be insufficient, or were stimulated too late in the course of the infection, for protection.

## Discussion

We examined the use of whole inactivated H5N1 virus as a post-exposure treatment against lethal homologous infection in mice. Mice infected with a lethal dose of V1203 and treated immediately with WI-V1203 were completely protected from infection and protection was dose dependent. Furthermore, immediate treatment after infection generated the best efficacy with some reduction in survival seen if treatment was delayed by 24 h and complete loss of efficacy with further delays in treatment to 2 and 3 days after infection.

Generally, vaccination with inactivated virus elicits a humoral immune response towards primarily the HA and NA surface glycoproteins. Antibodies directed against the globular head of HA can neutralize virus by inhibiting binding to host cellular receptors; HA specific antibodies are known to be the main correlate of protection^28^ provided by vaccination with an inactivated influenza vaccine. The adaptive immune response can take 4 to 7 days to activate; as a result, antigen-specific antibodies can take as long as 14 days after vaccination to be produced^29^. Mice infected with a low dose of H5N1 progress to disease slowly, taking 6 to 7 days before they show signs of clinical illness and succumb to infection by day 10. In this study, it is apparent that a protective adaptive immune response was being generated by administering a high dose treatment of whole virus inactivated vaccine soon after infection, and early enough to overcome the infection.

H5N1 virus infections in mice are known to replicate to high titers, spread systemically to multiple organs including the brain^11^,^30^, and cause high levels of CD8+ T cell activation^31^. As a result, H5N1 virus infection in the mouse is a highly stringent model to use for protection studies. In human cases of H5N1 virus infection, high levels of inflammatory cytokines and chemokines are associated with fatal disease outcomes^32^. In this study, T cell activation in the lung and spleen of mice from the (+)infected/(−)treated group was significantly elevated compared to mice from the (+)infected/(+)treated group. Results of the (+)infected/(−)treated mice agree with other studies that have shown that lethal H5N1 infection in mice can stimulate a strong CD8 T cell response in the lungs that is incapable of controlling viral replication^31^. Since less T cell activation is occurring in the (+)infected/(+)treated mice, it is possible that WI-V1203 treatment focused the immune response to be more Th-2 like, which is supported by the observation that a stronger and faster humoral immune response was induced by the (+)infected/(+)treated group with modulation of cellular responses. While these changes describe the magnitude of changes in T cell populations relative to the total lymphocyte population, it is important to note that because we did not measure absolute cell numbers, the percent changes of different T-cell subsets described do not quantify the magnitude of the response. Further determination of the magnitude of the T cell response may provide additional indication of treatment parameters that would provide optimal immune response and further evaluation in future studies is warranted.

Mice from the (+)infected/(+)treated group had consistently higher IgM and IgG titers at 8 dpi compared to the (+)infected/(−)treated group. Although antibodies were detected in the (+)infected/(−)treated group, associated neutralizing activity was not detected in contrast to the (+)infected/(+)treated group. The detection of nAbs in only the WI-V1203 treated group, even as early as 6 dpi, suggests that humoral immunity likely played a key role in protection. Although IgG is considered the dominant antibody that affords protection induced by vaccination, IgA also plays an important role in influenza viral clearance^33^,^34^. IgA is present in mucosal surfaces, and can neutralize viruses before they pass the mucosal barrier and enter the host^35^,^36^. Surprisingly, at 8 dpi, only the (+)infected/(+)treated group had any detectable level of IgA in the lungs. By 10 dpi, both (+)infected/(−)treated and (+)infected/(+)treated groups showed high IgA lung titers, suggesting that intranasal infection of mice was necessary to induce a strong IgA response compared to vaccination alone. Similar to lung IgG and IgM responses, the (+)infected/(+)treated mice had significantly higher titers of IgA compared to the (−)infected/(+)treated mice at 28 dpi. Therefore, it seems that treatment with WI-V1203 alone is only mildly immunogenic, and infection with V1203 acted synergistically with treatment to direct the immune response to faster and stronger humoral immunity leading to protection. Future experiments will need to include the quantification of Th-1 and Th-2 cytokines and chemokines released after infection and treatment with WI-V1203 to show if treatment is driving a stronger Th-2 response.

In this study, mice were infected IN which induces mucosal immunity, and then subsequently injected IM with WI-V1203. The dual exposure of both live and inactivated virus in these mice may be leading to greater and faster antigen presentation, resulting in rapid virus-specific antibody production and protective cellular responses. Considering the tight schedule between H5N1 infection and the production of protective antibodies, we were interested in determining the maximum delay for WI-V1203 treatment to still achieve protection. Survival dropped to 83.3% in mice that were treated with WI-V1203 at 1 dpi, and these mice had reduced viral titers in multiple organs and detectable levels of nAbs by 8 dpi in some animals. These nAbs were detected 2 days later compared to mice treated with WI-V1203 immediately after infection, linking the presence of the vaccine to enhanced humoral immunity, as mice from the (+)infected/(−)treated group did not generate any detectable nAbs. Further improvements to the WI-V1203 treatment such as inclusion of immunogenicity enhancing substances, like adjuvants, may help boost the immune system and lower the amount of antigen required for protection, and may also extend the window of opportunity to provide effective post-exposure treatment.

In summary, we have shown that whole virion inactivated H5N1 vaccines can be used as an effective post-exposure treatment against lethal H5N1 infection in mice, and treatment administered up to 1 day post-infection is still highly protective. Mice treated with WI-V1203 exhibited a diminished CD8 T cell response but a faster and stronger nAb response, which together improved the outcome of H5N1 infection. Here we present an alternative use of an inactivated influenza vaccine for post-exposure prophylaxis. These findings offer a novel strategy for the use of pre-manufactured H5N1 vaccines for pandemic preparedness, or vaccines that could be made in response to a novel H5N1 virus outbreak, as a post-exposure intervention. Although our study showed success against lethal homologous infection, an exact match between inactivated vaccines and potential outbreak strains is unlikely. Assessment of the efficacy of post-vaccination treatment against different clades of H5N1 will be an important next step to further support the use of this treatment strategy. Future studies with other animal models of influenza infection, as well as development of similar strategies for other high risk emerging influenza infection in humans such as the H7N9 virus that currently circulates in China, is warranted.

## Methods

### Cells and Viruses

Experiments with H5N1 viruses were all performed in BSL-4 containment. A/Vietnam/1203/2004 (H5N1) (V1203) virus stock was generated by reverse genetics and stocks were prepared by passage on Madin-Darby Canine Kidney (MDCK) (American Type Culture Collection) cells cultured with Minimal Essential Medium (MEM) supplemented with 0.1% bovine serum albumin (MEM/BSA) and 1 μg/ml TPCK-trypsin. To generate concentrated V1203 virus stocks, 10× T150 cm^2^ flasks of MDCK cells were infected with an MOI of 0.1 for 48 h. Supernatant from all flasks were combined, centrifuged for 15 min at 2500 rpm to pellet cell debris. Cleared supernatant was centrifuged at 25,000 rpm for 1 hr [SW32Ti rotor (Beckman Coulter] to pellet virus. Pellets were resuspended in 1 mL of PBS, an aliquot was removed for titration by plaque assay on MDCK cells. Concentrated V1203 was immediately inactivated by adding formalin to a final concentration of 0.1% in PBS and incubated at 4 °C for 3 days to produce whole-inactivated V1203 (WI-V1203). Safety testing of WI-V1203 was performed by plating a dilution series of the inactivated virus onto MDCK cells and observing for the absence of cytopathic effect in the wells 48–72 h later.

### Ethics Statement

All experiments on live animals were approved by the Canadian Science Centre for Human & Animal Health Animal Care Committee in protocol H-14-005. This protocol was carried out in accordance to the guidelines set by the Canadian Council on Animal Care.

### Mice

Female 4–6 week old BALB/c mice were purchased from Charles River Laboratories (Montreal, Quebec). Mice were housed in caging units that provide negative pressure and HEPA-filtered containment in the biosafety level 4 facilities (BSL-4) of the Public Health Agency of Canada–National Microbiology Laboratory. All mice were acclimatized for 7 days prior to the start of the experiment. The mouse lethal dose 50% (LD_50_) for V1203 was determined to be 1 PFU from previous data. Mice were infected IN with either 3LD_50_ (3 PFU) or 5LD_50_ (5 PFU) of V1203 in a volume of 50 μl virus diluent (MEM/0.1%BSA), or were mock-infected with virus diluent. Mice were treated post-exposure with different doses of WI-V1203 in a final volume of 50 μl by IM injection to the hind limb, or were mock-treated with 50 μl of PBS. Mice were monitored for clinical signs of disease and weight loss for 18 days, cut off for humane euthanasia was 25% weight loss.

### Virus titration and antibody assays

Pre-weighed tissues from the lung, spleen and kidney were harvested and homogenized for each treatment group using TissueLyser II (QIAGEN) according to manufacturer’s protocol. Replicating virus was measured from the homogenized supernatant by tissue culture infectious dose 50% (TCID_50_) assay on MDCK cells and titers per gram of tissue are reported. Levels of IgM, IgG, and IgA were determined in the serum and lung homogenates of mice by ELISA. Briefly, half-area 96-well plates were coated with 2 μg/ml of whole 0.1% formaldehyde-inactivated V1203 in PBS overnight at 4 °C. Plates were blocked with 5% skim milk in PBS for 1 hr at 37 °C, washed 6X with PBS/0.1%Tween20, incubated with serum/lung homogenate serial dilutions (1:3) for 1 hr at 37 °C, washed and incubated with secondary peroxidase conjugated anti-mouse IgM, IgG or IgA (KPL) for 1 hr at 37 °C, washed and developed with TMB solution (Life Technologies) for 30 min at RT. Cutoff values were determined as described by^37^ using control serum from 4 uninfected mice. Neutralizing antibodies were determined in the serum from mice by microneutralization assay. Serum was treated with receptor destroying enzyme (RDE) (Denka Seiken Co.) at 37 °C overnight, and inactivated for 60 min at 56 °C. Inactivated serum was serially diluted (1:2) and incubated with 100 PFU of virus for 1 hr at 37 °C. Virus and serum mixture was then added onto MDCK cells and infectivity was observed after 4 to 5 days at 37 °C.

### Flow cytometry

All antibodies used for flow cytometry were purchased from BD-Pharmingen. At specified time points, mice were sacrificed with an overdose of isoflurane and the lungs and spleen were dissected. Lung tissue was injected with collagenase D (Roche) and incubated at 37 °C for 30 min before being homogenized in RPMI media (GIBCO) through a 40 μm nylon mesh cell strainer with a syringe plunger, while spleen tissues were homogenized immediately. Cells were pelleted, resuspended in RPMI, and counted. Approximately 1 × 10^6^ cells were added to each well of a 96-well plate, 1 ml of PBS was added per well, and cells were centrifuged at 300 × g for 5 min at RT and stained. Cells were first stained with a viability dye containing Fc Block for 20 min at room temperature in the dark, and then further stained with anti-CD3, anti-CD4, anti-CD8 and anti-CD69 for 30 min at room temperature in the dark. Following staining, cells were fixed with Cytofix/Cytoperm (BD-Pharmingen) and removed from BSL-4 as described in PHAC-NML BSL-4 sample removal standard operating procedures. Samples were analyzed using a BD™ LSRII flow cytometer and data was analyzed using Flowjo software.

### Statistical analysis

All statistical analysis tests were performed using GraphPad Prism software. For virus titration, flow cytometry and ELISA assays (days 2 to 10), 2-way ANOVA analysis with Bonferroni post-tests were performed to determine statistical significance between mock-infected control mice [(−)infected/(+)treated] (n = 6), mock-treated mice [(+)infected/(−)treated] (n = 5 to 6), and infected and treated mice [(+)infected/(+)treated] (n = 6) at each time point. For ELISA results measured at the 28 dpi time-point, 2-sided unpaired t-tests with Welch’s correction were performed. Results were considered statistically significant when a minimum p value of <0.05 was attained.

## Additional Information

**Accession codes:** The Genbank Accession numbers for the gene segments of A/Vietnam/1203/2004 are AY818126 (PB2), AY818129 (PB1), AY818132 (PA), AY818135 (HA), AY818138 (NP), AY818141 (NA), AY818144 (M), AY818147 (NS).

**How to cite this article**: Hagan, M. *et al*. Post-exposure treatment with whole inactivated H5N1 avian influenza virus protects against lethal homologous virus infection in mice. *Sci. Rep.* **6**, 29433; doi: 10.1038/srep29433 (2016).

## Supplementary Material

Supplementary Information
