# Supplementary material for: Post-exposure treatment with whole inactivated H5N1 avian influenza virus protects against lethal homologous virus infection in mice
Source: Sci Rep. 2016 Jul 12;6:29433. doi: 10.1038/srep29433 (PMC4942574; doi:10.1038/srep29433)
Supplement: Supplementary Information [file srep29433-s1.doc]

Title: Post-exposure treatment with whole inactivated H5N1 avian influenza virus protects against lethal homologous virus infection in mice

Authors: Mable Hagan, Charlene Ranadheera, Jonathan Audet, Jocelyn Morin, Anders Leung, Darwyn Kobasa

**Supplementary Data. Individual lung and spleen total cell counts determined by flow cytometry.** Mice were infected IN with 3LD50 (3 PFU) of V1203 and treated with either WI-V1203 [(+)infected/(+)treated)] or PBS [(+)infected/(-)treated)] by IM injection. Uninfected and untreated mice were sacrificed on day 0 to determine baseline levels. As treatment controls, a group of mice were mock-infected with virus diluent and treated with WI-V1203 [(-)infected/(+)treated)]. Mice were sacrificed on day 2, 4, 6, 8, and 10 and lung and spleen tissue samples were removed, weighed, and the number of live cells/ml was determined. The total number of CD3+, CD3+CD4+, and CD3+CD8+ expressing T cells for each sample was measured, and CD4 and CD8 T cell activation was measured by CD69 expression. Based on the percent populations of each T cell subset determined, the total number of cells per gram of tissue (# cells/g) was calculated using the initial tissue sample weight measured.

| Group | Day/ Organ | Mouse | Weight (g) | Resus. Vol. (ml) | Live count (cells/ ml) | Total # cells | Total # cells/g |
| --- | --- | --- | --- | --- | --- | --- | --- |
| Uninfected/untreated | 0  Lung | 1 | 0.087 | 0.2 | 1770000 | 354000 | 4068966 |
|  |  | 2 | 0.15 | 0.2 | 5240000 | 1048000 | 6986667 |
|  |  | 3 | 0.081 | 0.2 | 2250000 | 450000 | 5555556 |
|  |  | 4 | 0.237 | 0.2 | 2480000 | 496000 | 2092827 |

| Group | Day/ Organ | Mouse | Total events | Singlets | Lympho-cytes | CD3 | CD4 | CD4 CD69 | CD8 | CD8 CD69 |
| --- | --- | --- | --- | --- | --- | --- | --- | --- | --- | --- |
| Uninfected/ untreated | 0  Lung | 1 | 24738 | 22486 | 16450 | 7501 | 4803 | 44 | 2293 | 24 |
|  |  | 2 | 94468 | 83136 | 60656 | 27053 | 18013 | 218 | 8102 | 169 |
|  |  | 3 | 29499 | 25459 | 16721 | 7076 | 4735 | 36 | 2094 | 13 |
|  |  | 4 | 35283 | 30896 | 21227 | 9455 | 6225 | 45 | 2989 | 26 |

| Group | Day/ Organ | Mouse | %  Lymphocytes | Total # lymphoctyes/g | % CD3 | Total # CD3/g | % CD4 | Total # CD4/g |
| --- | --- | --- | --- | --- | --- | --- | --- | --- |
| Uninfected/ untreated | 0  Lung | 1 | 73 | 2976718 | 46 | 1357347 | 64 | 869129 |
|  |  | 2 | 73 | 5097470 | 45 | 2273507 | 67 | 1513795 |
|  |  | 3 | 66 | 3648786 | 42 | 1544095 | 67 | 1033252 |
|  |  | 4 | 69 | 1437870 | 45 | 640461 | 66 | 421668 |

| Group | Day/ Organ | Mouse | % CD4  CD69 | Total # CD4CD69/g | | % CD8 | | Total # CD8/g | | % CD8CD69 | Total # CD8CD69/g | |
| --- | --- | --- | --- | --- | --- | --- | --- | --- | --- | --- | --- | --- |
| Uninfected/ untreated | 0  Lung | 1 | 1 | 7962 | | 31 | | 414931 | | 1 | 4343 | |
|  |  | 2 | 1 | 18321 | | 30 | | 680884 | | 2 | 14203 | |
|  |  | 3 | 1 | 7856 | | 30 | | 456944 | | 1 | 2837 | |
|  |  | 4 | 1 | 3048 | | 32 | | 202468 | | 1 | 1761 | |
| Group | Day/ Organ | Mouse | Weight (g) | | Resus. Vol. (ml) | | Live count (cells/ ml) | | Total # cells | | | Total # cells/g |
| (-)infected/ (+)treated | 2  Lung | 1 | 0.191 | | 0.15 | | 1940000 | | 291000 | | | 1523560 |
|  |  | 2 | 0.236 | | 0.15 | | 1700000 | | 255000 | | | 1080508 |
|  |  | 3 | 0.187 | | 0.15 | | 1920000 | | 288000 | | | 1540107 |
|  |  | 4 | 0.205 | | 0.15 | | 1380000 | | 207000 | | | 1009756 |
|  |  | 5 | 0.063 | | 0.15 | | 2270000 | | 340500 | | | 5404762 |
|  |  | 6 | 0.165 | | 0.15 | | 2280000 | | 342000 | | | 2072727 |
| (+)infected/(+)treated | 2  Lung | 1 | 0.253 | | 0.15 | | 1400000 | | 210000 | | | 830040 |
|  |  | 2 | 0.076 | | 0.15 | | 1620000 | | 243000 | | | 3197368 |
|  |  | 3 | 0.063 | | 0.15 | | 2030000 | | 304500 | | | 4833333 |
|  |  | 4 | 0.25 | | 0.15 | | 4180000 | | 627000 | | | 2508000 |
|  |  | 5 | 0.101 | | 0.15 | | 2840000 | | 426000 | | | 4217822 |
|  |  | 6 | 0.071 | | 0.15 | | 1650000 | | 247500 | | | 3485915 |
| (+)infected/(-)treated | 2  Lung | 1 | 0.071 | | 0.15 | | 3320000 | | 498000 | | | 7014085 |
|  |  | 2 | 0.231 | | 0.15 | | 1960000 | | 294000 | | | 1272727 |
|  |  | 3 | 0.036 | | 0.15 | | 1850000 | | 277500 | | | 7708333 |
|  |  | 4 | 0.183 | | 0.15 | | 2530000 | | 379500 | | | 2073770 |
|  |  | 5 | 0.228 | | 0.15 | | 1610000 | | 241500 | | | 1059211 |
|  |  | 6 | 0.055 | | 0.15 | | 1570000 | | 235500 | | | 4281818 |

| Group | Day/ Organ | Mouse | Total events | Singlets | Lympho-  cytes | CD3 | CD4 | CD4 CD69 | CD8 | CD8 CD69 |
| --- | --- | --- | --- | --- | --- | --- | --- | --- | --- | --- |
| (-)infected/ (+)treated | 2  Lung | 1 | 48546 | 43907 | 23092 | 7841 | 5614 | 41 | 1758 | 23 |
|  |  | 2 | 42734 | 38859 | 21247 | 9831 | 7019 | 69 | 2297 | 36 |
|  |  | 3 | 38628 | 35097 | 21893 | 9977 | 7026 | 85 | 2497 | 29 |
|  |  | 4 | 25252 | 23765 | 16416 | 8270 | 5995 | 127 | 2000 | 39 |
|  |  | 5 | 54813 | 50955 | 24193 | 8322 | 5699 | 126 | 2124 | 28 |
|  |  | 6 | 51797 | 47182 | 30174 | 14579 | 10543 | 138 | 3454 | 58 |
| (+)infected/ (+)treated | 2  Lung | 1 | 36234 | 32825 | 19324 | 6939 | 4710 | 65 | 1954 | 46 |
|  |  | 2 | 32450 | 28927 | 17834 | 7984 | 5607 | 73 | 2035 | 38 |
|  |  | 3 | 34867 | 31584 | 20707 | 9705 | 6832 | 134 | 2442 | 55 |
|  |  | 4 | 87740 | 76281 | 43150 | 19224 | 12499 | 211 | 5258 | 187 |
|  |  | 5 | 64765 | 56588 | 33653 | 15702 | 10991 | 212 | 3942 | 106 |
|  |  | 6 | 27578 | 24944 | 14138 | 6137 | 4288 | 134 | 1520 | 61 |
| (+)infected/ (-)treated | 2  Lung | 1 | 79099 | 72897 | 44103 | 20398 | 14175 | 50 | 5464 | 48 |
|  |  | 2 | 46595 | 42388 | 28004 | 15475 | 10853 | 64 | 4084 | 40 |
|  |  | 3 | 46057 | 42169 | 26562 | 11618 | 8021 | 59 | 3180 | 35 |
|  |  | 4 | 61859 | 56188 | 34973 | 17580 | 12016 | 151 | 4677 | 231 |
|  |  | 5 | 37338 | 33316 | 18425 | 7580 | 5215 | 32 | 2131 | 19 |
|  |  | 6 | 45439 | 41744 | 25573 | 12118 | 8659 | 135 | 2995 | 119 |

| Group | Day/ Organ | Mouse | %  Lymphocytes | Total # lymphoctyes/g | % CD3 | Total #CD3/g | % CD4 | Total # CD4/g |
| --- | --- | --- | --- | --- | --- | --- | --- | --- |
| (-)infected/ (+)treated | 2  Lung | 1 | 53 | 801286 | 34 | 272080 | 72 | 194804 |
|  |  | 2 | 55 | 590791 | 46 | 273360 | 71 | 195169 |
|  |  | 3 | 62 | 960696 | 46 | 437805 | 70 | 308311 |
|  |  | 4 | 69 | 697503 | 50 | 351386 | 72 | 254723 |
|  |  | 5 | 47 | 2566135 | 34 | 882709 | 68 | 604489 |
|  |  | 6 | 64 | 1325558 | 48 | 640462 | 72 | 463159 |
| (+)infected/ (+)treated | 2  Lung | 1 | 59 | 488642 | 36 | 175465 | 68 | 119101 |
|  |  | 2 | 62 | 1971233 | 45 | 882490 | 70 | 619755 |
|  |  | 3 | 66 | 3168814 | 47 | 1485167 | 70 | 1045508 |
|  |  | 4 | 57 | 1418705 | 45 | 632055 | 65 | 410948 |
|  |  | 5 | 59 | 2508347 | 47 | 1170358 | 70 | 819221 |
|  |  | 6 | 57 | 1975781 | 43 | 857644 | 70 | 599247 |
| (+)infected/ (-)treated | 2  Lung | 1 | 61 | 4243551 | 46 | 1962677 | 69 | 1363906 |
|  |  | 2 | 66 | 840838 | 55 | 464647 | 70 | 325868 |
|  |  | 3 | 63 | 4855433 | 44 | 2123726 | 69 | 1466208 |
|  |  | 4 | 62 | 1290773 | 50 | 648838 | 68 | 443483 |
|  |  | 5 | 55 | 585783 | 41 | 240990 | 69 | 165800 |
|  |  | 6 | 61 | 2623106 | 47 | 1242983 | 71 | 888182 |

| Group | Day/ Organ | Mouse | % CD4  CD69 | Total # CD4CD69/g | % CD8 | Total # CD8/g | % CD8CD69 | Total # CD8CD69/g |
| --- | --- | --- | --- | --- | --- | --- | --- | --- |
| (-)infected/ (+)treated | 2  Lung | 1 | 1 | 1423 | 22 | 61002 | 1 | 798 |
|  |  | 2 | 1 | 1919 | 23 | 63870 | 2 | 1001 |
|  |  | 3 | 1 | 3730 | 25 | 109572 | 1 | 1273 |
|  |  | 4 | 2 | 5396 | 24 | 84978 | 2 | 1657 |
|  |  | 5 | 2 | 13365 | 26 | 225291 | 1 | 2970 |
|  |  | 6 | 1 | 6062 | 24 | 151736 | 2 | 2548 |
| (+)infected/ (+)treated | 2  Lung | 1 | 1 | 1644 | 28 | 49410 | 2 | 1163 |
|  |  | 2 | 1 | 8069 | 25 | 224933 | 2 | 4200 |
|  |  | 3 | 2 | 20506 | 25 | 373702 | 2 | 8417 |
|  |  | 4 | 2 | 6937 | 27 | 172875 | 4 | 6148 |
|  |  | 5 | 2 | 15802 | 25 | 293819 | 3 | 7901 |
|  |  | 6 | 3 | 18726 | 25 | 212419 | 4 | 8525 |
| (+)infected/ (-)treated | 2  Lung | 1 | 0 | 4811 | 27 | 525741 | 1 | 4619 |
|  |  | 2 | 1 | 1922 | 26 | 122625 | 1 | 1201 |
|  |  | 3 | 1 | 10785 | 27 | 581292 | 1 | 6398 |
|  |  | 4 | 1 | 5573 | 27 | 172617 | 5 | 8526 |
|  |  | 5 | 1 | 1017 | 28 | 67751 | 1 | 604 |
|  |  | 6 | 2 | 13847 | 25 | 307207 | 4 | 12206 |

| Group | Day/ Organ | Mouse | Weight (g) | Resus. Vol. (ml) | Live count (cells/ ml) | Total # cells | Total # cells/g |
| --- | --- | --- | --- | --- | --- | --- | --- |
| (-)infected/ (+)treated | 4  Lung | 1 | 0.079 | 0.15 | 2730000 | 409500 | 5183544 |
|  |  | 2 | 0.076 | 0.15 | 3000000 | 450000 | 5921053 |
|  |  | 3 | 0.078 | 0.15 | 2990000 | 448500 | 5750000 |
|  |  | 4 | 0.114 | 0.15 | 4200000 | 630000 | 5526316 |
|  |  | 5 | 0.076 | 0.15 | 5000000 | 750000 | 9868421 |
|  |  | 6 | 0.083 | 0.15 | 3220000 | 483000 | 5819277 |
| (+)infected/(+)treated | 4  Lung | 1 | 0.085 | 0.15 | 5630000 | 844500 | 9935294 |
|  |  | 2 | 0.101 | 0.15 | 8240000 | 1236000 | 12237624 |
|  |  | 3 | 0.1 | 0.15 | 5130000 | 769500 | 7695000 |
|  |  | 4 | 0.084 | 0.15 | 6400000 | 960000 | 11428571 |
|  |  | 5 | 0.077 | 0.15 | 4470000 | 670500 | 8707792 |
|  |  | 6 | 0.084 | 0.15 | 5240000 | 786000 | 9357143 |
| (+)infected/(-)treated | 4  Lung | 1 | 0.074 | 0.15 | 3740000 | 561000 | 7581081 |
|  |  | 2 | 0.093 | 0.15 | 10600000 | 1590000 | 17096774 |
|  |  | 3 | 0.105 | 0.15 | 10500000 | 1575000 | 15000000 |
|  |  | 4 | 0.112 | 0.15 | 6970000 | 1045500 | 9334821 |
|  |  | 5 | 0.098 | 0.15 | 5820000 | 873000 | 8908163 |
|  |  | 6 | 0.102 | 0.15 | 10600000 | 1590000 | 15588235 |

| Group | Day/ Organ | Mouse | Total events | Singlets | Lympho-  cytes | CD3 | CD4 | CD4 CD69 | CD8 | CD8 CD69 |
| --- | --- | --- | --- | --- | --- | --- | --- | --- | --- | --- |
| (-)infected/ (+)treated | 4  Lung | 1 | 64473 | 59260 | 33986 | 13457 | 7892 | 201 | 3903 | 581 |
|  |  | 2 | 57094 | 52205 | 35114 | 13364 | 8473 | 46 | 4020 | 26 |
|  |  | 3 | 71427 | 63173 | 38669 | 14345 | 9819 | 48 | 3980 | 26 |
|  |  | 4 | 76483 | 69669 | 37294 | 13832 | 7766 | 110 | 3934 | 345 |
|  |  | 5 | 115579 | 107308 | 73569 | 36295 | 20500 | 1144 | 10127 | 445 |
|  |  | 6 | 76293 | 71304 | 42575 | 12975 | 8570 | 132 | 3319 | 48 |
| (+)infected/ (+)treated | 4  Lung | 1 | 64987 | 54716 | 32183 | 14336 | 9869 | 334 | 3479 | 365 |
|  |  | 2 | 88970 | 73893 | 42085 | 16717 | 9179 | 218 | 4091 | 416 |
|  |  | 3 | 83736 | 70931 | 41953 | 14799 | 7412 | 168 | 3840 | 274 |
|  |  | 4 | 104848 | 85874 | 43650 | 15603 | 9613 | 319 | 3211 | 334 |
|  |  | 5 | 113315 | 93741 | 54279 | 19013 | 12152 | 218 | 4904 | 278 |
|  |  | 6 | 78497 | 67596 | 29561 | 8699 | 5877 | 258 | 1776 | 214 |
| (+)infected/ (-)treated | 4  Lung | 1 | 64518 | 53594 | 32348 | 12160 | 8735 | 169 | 2715 | 198 |
|  |  | 2 | 135223 | 110642 | 56340 | 23691 | 14840 | 599 | 5433 | 990 |
|  |  | 3 | 172656 | 137133 | 77148 | 39015 | 28199 | 762 | 8400 | 926 |
|  |  | 4 | 172561 | 131991 | 60572 | 21702 | 15274 | 453 | 4496 | 575 |
|  |  | 5 | 103920 | 82131 | 44103 | 17308 | 12466 | 436 | 3826 | 362 |
|  |  | 6 | 252548 | 195005 | 89674 | 34529 | 24388 | 1077 | 7551 | 1139 |

| Group | Day/ Organ | Mouse | %  Lymphocytes | Total # lymphoctyes/g | % CD3 | Total #CD3/g | % CD4 | Total # CD4/g |
| --- | --- | --- | --- | --- | --- | --- | --- | --- |
| (-)infected/ (+)treated | 4  Lung | 1 | 57 | 2972797 | 40 | 1177100 | 59 | 690323 |
|  |  | 2 | 67 | 3982604 | 38 | 1515735 | 63 | 961001 |
|  |  | 3 | 61 | 3519648 | 37 | 1305680 | 68 | 893724 |
|  |  | 4 | 54 | 2958251 | 37 | 1097188 | 56 | 616018 |
|  |  | 5 | 69 | 6765664 | 49 | 3337816 | 56 | 1885252 |
|  |  | 6 | 60 | 3474640 | 30 | 1058918 | 66 | 699417 |
| (+)infected/ (+)treated | 4  Lung | 1 | 59 | 5843767 | 45 | 2603121 | 69 | 1792006 |
|  |  | 2 | 57 | 6969813 | 40 | 2768549 | 55 | 1520160 |
|  |  | 3 | 59 | 4551301 | 35 | 1605480 | 50 | 804096 |
|  |  | 4 | 51 | 5809176 | 36 | 2076531 | 62 | 1279349 |
|  |  | 5 | 58 | 5042087 | 35 | 1766156 | 64 | 1128824 |
|  |  | 6 | 44 | 4092054 | 29 | 1204181 | 68 | 813538 |
| (+)infected/ (-)treated | 4  Lung | 1 | 60 | 4575751 | 38 | 1720080 | 72 | 1235600 |
|  |  | 2 | 51 | 8705846 | 42 | 3660813 | 63 | 2293127 |
|  |  | 3 | 56 | 8438669 | 51 | 4267572 | 72 | 3084487 |
|  |  | 4 | 46 | 4283844 | 36 | 1534834 | 70 | 1080226 |
|  |  | 5 | 54 | 4783538 | 39 | 1877275 | 72 | 1352098 |
|  |  | 6 | 46 | 7168326 | 39 | 2760166 | 71 | 1949519 |

| Group | Day/ Organ | Mouse | % CD4  CD69 | Total # CD4CD69/g | % CD8 | Total # CD8/g | % CD8CD69 | Total # CD8CD69/g |
| --- | --- | --- | --- | --- | --- | --- | --- | --- |
| (-)infected/ (+)treated | 4  Lung | 1 | 3 | 17582 | 22 | 61002 | 1 | 798 |
|  |  | 2 | 1 | 5217 | 23 | 63870 | 2 | 1001 |
|  |  | 3 | 0 | 4369 | 25 | 109572 | 1 | 1273 |
|  |  | 4 | 1 | 8725 | 24 | 84978 | 2 | 1657 |
|  |  | 5 | 6 | 105206 | 26 | 225291 | 1 | 2970 |
|  |  | 6 | 2 | 10773 | 24 | 151736 | 2 | 2548 |
| (+)infected/ (+)treated | 4  Lung | 1 | 3 | 60647 | 28 | 49410 | 2 | 1163 |
|  |  | 2 | 2 | 36104 | 25 | 224933 | 2 | 4200 |
|  |  | 3 | 2 | 18226 | 25 | 373702 | 2 | 8417 |
|  |  | 4 | 3 | 42454 | 27 | 172875 | 4 | 6148 |
|  |  | 5 | 2 | 20250 | 25 | 293819 | 3 | 7901 |
|  |  | 6 | 4 | 35714 | 25 | 212419 | 4 | 8525 |
| (+)infected/ (-)treated | 4  Lung | 1 | 2 | 23906 | 27 | 525741 | 1 | 4619 |
|  |  | 2 | 4 | 92559 | 26 | 122625 | 1 | 1201 |
|  |  | 3 | 3 | 83350 | 27 | 581292 | 1 | 6398 |
|  |  | 4 | 3 | 32038 | 27 | 172617 | 5 | 8526 |
|  |  | 5 | 3 | 47290 | 28 | 67751 | 1 | 604 |
|  |  | 6 | 4 | 86093 | 25 | 307207 | 4 | 12206 |

| Group | Day/ Organ | Mouse | Weight (g) | Resus. Vol. (ml) | Live count (cells/ ml) | Total # cells | Total # cells/g |
| --- | --- | --- | --- | --- | --- | --- | --- |
| (-)infected/ (+)treated | 6  Lung | 1 | 0.093 | 0.15 | 6610000 | 991500 | 10661290 |
|  |  | 2 | 0.101 | 0.15 | 6090000 | 913500 | 9044554 |
|  |  | 3 | 0.073 | 0.15 | 7230000 | 1084500 | 14856164 |
|  |  | 4 | 0.077 | 0.15 | 2770000 | 415500 | 5396104 |
|  |  | 5 | 0.109 | 0.15 | 2980000 | 447000 | 4100917 |
|  |  | 6 | 0.074 | 0.15 | 2170000 | 325500 | 4398649 |
| (+)infected/(+)treated | 6  Lung | 1 | 0.11 | 0.15 | 13800000 | 2070000 | 18818182 |
|  |  | 2 | 0.084 | 0.15 | 7700000 | 1155000 | 13750000 |
|  |  | 3 | 0.115 | 0.15 | 23400000 | 3510000 | 30521739 |
|  |  | 4 | 0.115 | 0.15 | 15700000 | 2355000 | 20478261 |
|  |  | 5 | 0.11 | 0.15 | 18500000 | 2775000 | 25227273 |
|  |  | 6 | 0.105 | 0.15 | 13900000 | 2085000 | 19857143 |
| (+)infected/(-)treated | 6  Lung | 1 | 0.136 | 0.15 | 21100000 | 3165000 | 23272059 |
|  |  | 2 | 0.118 | 0.15 | 12500000 | 1875000 | 15889831 |
|  |  | 3 | 0.106 | 0.15 | 12000000 | 1800000 | 16981132 |
|  |  | 4 | 0.122 | 0.15 | 12500000 | 1875000 | 15368852 |
|  |  | 5 | 0.1 | 0.15 | 10700000 | 1605000 | 16050000 |
|  |  | 6 | 0.111 | 0.15 | 9600000 | 1440000 | 12972973 |

| Group | Day/ Organ | Mouse | Total events | Singlets | Lympho-  cytes | CD3 | CD4 | CD4 CD69 | CD8 | CD8 CD69 |
| --- | --- | --- | --- | --- | --- | --- | --- | --- | --- | --- |
| (-)infected/ (+)treated | 6  Lung | 1 | 142540 | 127430 | 76694 | 28855 | 19954 | 118 | 7792 | 200 |
|  |  | 2 | 122006 | 107811 | 68614 | 27545 | 19369 | 122 | 7224 | 65 |
|  |  | 3 | 112282 | 96642 | 52478 | 21124 | 14575 | 930 | 4568 | 642 |
|  |  | 4 | 62375 | 53203 | 31821 | 13843 | 10020 | 63 | 3368 | 33 |
|  |  | 5 | 78594 | 67516 | 35604 | 16400 | 11572 | 189 | 4093 | 156 |
|  |  | 6 | 42018 | 36273 | 22656 | 10647 | 7873 | 55 | 2443 | 26 |
| (+)infected/ (+)treated | 6  Lung | 1 | 217588 | 181816 | 77607 | 26126 | 14829 | 1620 | 5277 | 909 |
|  |  | 2 | 125564 | 108609 | 64111 | 27407 | 16782 | 841 | 7699 | 1033 |
|  |  | 3 | 341923 | 284844 | 155609 | 60429 | 29581 | 3081 | 16642 | 3825 |
|  |  | 4 | 212078 | 176487 | 90684 | 32658 | 19807 | 2099 | 8267 | 1699 |
|  |  | 5 | 268393 | 228235 | 120335 | 46763 | 26667 | 2877 | 13018 | 2836 |
|  |  | 6 | 211193 | 173140 | 85905 | 31807 | 20154 | 1796 | 8310 | 1473 |
| (+)infected/ (-)treated | 6  Lung | 1 | 389499 | 303061 | 136240 | 32068 | 16257 | 597 | 8561 | 1866 |
|  |  | 2 | 242716 | 189756 | 94356 | 36095 | 21988 | 851 | 8521 | 1489 |
|  |  | 3 | 216346 | 175807 | 105358 | 42844 | 28711 | 1065 | 11518 | 1496 |
|  |  | 4 | 189880 | 152465 | 63028 | 19468 | 11928 | 393 | 4798 | 708 |
|  |  | 5 | 203627 | 160605 | 83587 | 26813 | 17397 | 572 | 6557 | 957 |
|  |  | 6 | 228540 | 178629 | 74695 | 23089 | 11521 | 473 | 5334 | 851 |

| Group | Day/ Organ | Mouse | %  Lymphocytes | Total # lymphoctyes/g | % CD3 | Total #CD3/g | % CD4 | Total # CD4/g |
| --- | --- | --- | --- | --- | --- | --- | --- | --- |
| (-)infected/ (+)treated | 6  Lung | 1 | 60 | 6416519 | 38 | 2414122 | 69 | 1669429 |
|  |  | 2 | 64 | 5756213 | 40 | 2310824 | 70 | 1624917 |
|  |  | 3 | 54 | 8067112 | 40 | 3247259 | 69 | 2240523 |
|  |  | 4 | 60 | 3227439 | 44 | 1404024 | 72 | 1016277 |
|  |  | 5 | 53 | 2162585 | 46 | 996135 | 71 | 702883 |
|  |  | 6 | 62 | 2747382 | 47 | 1291109 | 74 | 954720 |
| (+)infected/ (+)treated | 6  Lung | 1 | 43 | 8032421 | 34 | 2704073 | 57 | 1534820 |
|  |  | 2 | 59 | 8116512 | 43 | 3469752 | 61 | 2124617 |
|  |  | 3 | 55 | 16673889 | 39 | 6475117 | 49 | 3169677 |
|  |  | 4 | 51 | 10522308 | 36 | 3789395 | 61 | 2298259 |
|  |  | 5 | 53 | 13300869 | 39 | 5168808 | 57 | 2947557 |
|  |  | 6 | 50 | 9852304 | 37 | 3647893 | 63 | 2311429 |
| (+)infected/ (-)treated | 6  Lung | 1 | 45 | 10461872 | 24 | 2462502 | 51 | 1248375 |
|  |  | 2 | 50 | 7901204 | 38 | 3022531 | 61 | 1841236 |
|  |  | 3 | 60 | 10176490 | 41 | 4138286 | 67 | 2773185 |
|  |  | 4 | 41 | 6353380 | 31 | 1962423 | 61 | 1202372 |
|  |  | 5 | 52 | 8353235 | 32 | 2679547 | 65 | 1738563 |
|  |  | 6 | 42 | 5424742 | 31 | 1676844 | 50 | 836715 |

| Group | Day/ Organ | Mouse | % CD4  CD69 | Total # CD4CD69/g | % CD8 | Total # CD8/g | % CD8CD69 | Total # CD8CD69/g |
| --- | --- | --- | --- | --- | --- | --- | --- | --- |
| (-)infected/ (+)treated | 6  Lung | 1 | 1 | 9872 | 27 | 651909 | 3 | 16733 |
|  |  | 2 | 1 | 10235 | 26 | 606041 | 1 | 5453 |
|  |  | 3 | 6 | 142963 | 22 | 702210 | 14 | 98691 |
|  |  | 4 | 1 | 6390 | 24 | 341599 | 1 | 3347 |
|  |  | 5 | 2 | 11480 | 25 | 248609 | 4 | 9475 |
|  |  | 6 | 1 | 6670 | 23 | 296251 | 1 | 3153 |
| (+)infected/ (+)treated | 6  Lung | 1 | 11 | 167672 | 20 | 546176 | 17 | 94083 |
|  |  | 2 | 5 | 106471 | 28 | 974701 | 13 | 130779 |
|  |  | 3 | 10 | 330137 | 28 | 1783231 | 23 | 409858 |
|  |  | 4 | 11 | 243553 | 25 | 959242 | 21 | 197140 |
|  |  | 5 | 11 | 318001 | 28 | 1438906 | 22 | 313469 |
|  |  | 6 | 9 | 205980 | 26 | 953060 | 18 | 168936 |
| (+)infected/ (-)treated | 6  Lung | 1 | 4 | 45844 | 27 | 657399 | 22 | 143290 |
|  |  | 2 | 4 | 71261 | 24 | 713533 | 17 | 124686 |
|  |  | 3 | 4 | 102868 | 27 | 1112519 | 13 | 144498 |
|  |  | 4 | 3 | 39615 | 25 | 483650 | 15 | 71368 |
|  |  | 5 | 3 | 57163 | 24 | 655271 | 15 | 95637 |
|  |  | 6 | 4 | 34352 | 23 | 387383 | 16 | 61804 |

| Group | Day/ Organ | Mouse | Weight (g) | Resus. Vol. (ml) | Live count (cells/ ml) | Total # cells | Total # cells/g |
| --- | --- | --- | --- | --- | --- | --- | --- |
| (-)infected/ (+)treated | 8  Lung | 1 | 0.069 | 0.15 | 2050000 | 307500 | 4456522 |
|  |  | 2 | 0.045 | 0.15 | 1960000 | 294000 | 6533333 |
|  |  | 3 | 0.049 | 0.15 | 2460000 | 369000 | 7530612 |
|  |  | 4 | 0.074 | 0.15 | 2690000 | 403500 | 5452703 |
|  |  | 5 | 0.075 | 0.15 | 1480000 | 222000 | 2960000 |
|  |  | 6 | 0.096 | 0.15 | 1970000 | 295500 | 3078125 |
| (+)infected/(+)treated | 8  Lung | 1 | 0.119 | 0.15 | 6210000 | 931500 | 7827731 |
|  |  | 2 | 0.102 | 0.15 | 3390000 | 508500 | 4985294 |
|  |  | 3 | 0.101 | 0.15 | 6430000 | 964500 | 9549505 |
|  |  | 4 | 0.102 | 0.15 | 7170000 | 1075500 | 10544118 |
|  |  | 5 | 0.107 | 0.15 | 14600000 | 2190000 | 20467290 |
|  |  | 6 | 0.091 | 0.15 | 5490000 | 823500 | 9049451 |
| (+)infected/(-)treated | 8  Lung | 1 | 0.145 | 0.15 | 8080000 | 1212000 | 8358621 |
|  |  | 2 | 0.144 | 0.15 | 13000000 | 1950000 | 13541667 |
|  |  | 3 | 0.139 | 0.15 | 15400000 | 2310000 | 16618705 |
|  |  | 4 | 0.095 | 0.15 | 4360000 | 654000 | 6884211 |
|  |  | 5 | 0.133 | 0.15 | 13100000 | 1965000 | 14774436 |
|  |  | 6 | 0.126 | 0.15 | 7320000 | 1098000 | 8714286 |

| Group | Day/ Organ | Mouse | Total events | Singlets | Lympho-cytes | CD3 | CD4 | CD4 CD69 | CD8 | CD8 CD69 |
| --- | --- | --- | --- | --- | --- | --- | --- | --- | --- | --- |
| (-)infected/ (+)treated | 8  Lung | 1 | 43687 | 38877 | 26869 | 12851 | 8980 | 37 | 3370 | 37 |
|  |  | 2 | 45841 | 40912 | 25499 | 11078 | 7642 | 27 | 2825 | 19 |
|  |  | 3 | 68672 | 59847 | 35208 | 14300 | 8484 | 48 | 3979 | 84 |
|  |  | 4 | 82257 | 73427 | 47840 | 22017 | 12771 | 330 | 5923 | 145 |
|  |  | 5 | 39808 | 34006 | 20037 | 8874 | 5794 | 20 | 2350 | 17 |
|  |  | 6 | 57995 | 51466 | 32393 | 14338 | 7341 | 43 | 3910 | 99 |
| (+)infected/ (+)treated | 8  Lung | 1 | 223628 | 195660 | 112163 | 36596 | 16704 | 1021 | 11382 | 1687 |
|  |  | 2 | 92622 | 81439 | 49147 | 23279 | 7621 | 877 | 6009 | 1254 |
|  |  | 3 | 152921 | 136407 | 87428 | 43467 | 16698 | 1334 | 11511 | 2556 |
|  |  | 4 | 156114 | 136262 | 86508 | 41508 | 16253 | 1146 | 10518 | 1746 |
|  |  | 5 | 318533 | 278900 | 169844 | 84714 | 31656 | 3632 | 24769 | 7171 |
|  |  | 6 | 106253 | 94269 | 60572 | 29266 | 8669 | 633 | 8563 | 1650 |
| (+)infected/ (-)treated | 8  Lung | 1 | 267769 | 231555 | 118232 | 40947 | 10813 | 1699 | 12557 | 4452 |
|  |  | 2 | 399008 | 340337 | 164250 | 42004 | 18551 | 1506 | 12392 | 2409 |
|  |  | 3 | 378732 | 321423 | 153748 | 54559 | 16107 | 3227 | 18007 | 6996 |
|  |  | 4 | 89804 | 75670 | 34197 | 9413 | 4217 | 520 | 2606 | 744 |
|  |  | 5 | 194281 | 165319 | 82591 | 29312 | 9287 | 1293 | 9398 | 3178 |
|  |  | 6 | 157470 | 134243 | 63814 | 24379 | 8373 | 1346 | 7622 | 3209 |

| Group | Day/ Organ | Mouse | %  Lymphocytes | Total # lymphoctyes/g | % CD3 | Total #CD3/g | % CD4 | Total # CD4/g |
| --- | --- | --- | --- | --- | --- | --- | --- | --- |
| (-)infected/ (+)treated | 8  Lung | 1 | 69 | 3080029 | 48 | 1473127 | 70 | 1029389 |
|  |  | 2 | 62 | 4071995 | 43 | 1769072 | 69 | 1220369 |
|  |  | 3 | 59 | 4430260 | 41 | 1799384 | 59 | 1067551 |
|  |  | 4 | 65 | 3552607 | 46 | 1634987 | 58 | 948377 |
|  |  | 5 | 59 | 1744090 | 44 | 772424 | 65 | 504330 |
|  |  | 6 | 63 | 1937390 | 44 | 857540 | 51 | 439057 |
| (+)infected/ (+)treated | 8  Lung | 1 | 57 | 4487283 | 33 | 1464089 | 46 | 668274 |
|  |  | 2 | 60 | 3008537 | 47 | 1425026 | 33 | 466520 |
|  |  | 3 | 64 | 6120611 | 50 | 3043013 | 38 | 1168984 |
|  |  | 4 | 63 | 6694093 | 48 | 3211939 | 39 | 1257677 |
|  |  | 5 | 61 | 12464132 | 50 | 6216802 | 37 | 2323100 |
|  |  | 6 | 64 | 5814672 | 48 | 2809420 | 30 | 832190 |
| (+)infected/ (-)treated | 8  Lung | 1 | 51 | 4267912 | 35 | 1478096 | 26 | 390325 |
|  |  | 2 | 48 | 6535342 | 26 | 1671297 | 44 | 738126 |
|  |  | 3 | 48 | 7949315 | 35 | 2820893 | 30 | 832789 |
|  |  | 4 | 45 | 3111132 | 28 | 856364 | 45 | 383649 |
|  |  | 5 | 50 | 7381096 | 35 | 2619592 | 32 | 829972 |
|  |  | 6 | 48 | 4142439 | 38 | 1582545 | 34 | 543527 |

| Group | Day/ Organ | Mouse | % CD4  CD69 | Total # CD4CD69/g | % CD8 | Total # CD8/g | % CD8CD69 | Total # CD8CD69/g |
| --- | --- | --- | --- | --- | --- | --- | --- | --- |
| (-)infected/ (+)treated | 8  Lung | 1 | 0 | 4241 | 26 | 386308 | 1 | 4241 |
|  |  | 2 | 0 | 4312 | 26 | 451131 | 1 | 3034 |
|  |  | 3 | 1 | 6040 | 28 | 500682 | 2 | 10570 |
|  |  | 4 | 3 | 24506 | 27 | 439843 | 2 | 10768 |
|  |  | 5 | 0 | 1741 | 26 | 204552 | 1 | 1480 |
|  |  | 6 | 1 | 2572 | 27 | 233853 | 3 | 5921 |
| (+)infected/ (+)treated | 8  Lung | 1 | 6 | 40847 | 31 | 455357 | 15 | 67491 |
|  |  | 2 | 12 | 53686 | 26 | 367841 | 21 | 76764 |
|  |  | 3 | 8 | 93390 | 26 | 805856 | 22 | 178939 |
|  |  | 4 | 7 | 88679 | 25 | 813896 | 17 | 135108 |
|  |  | 5 | 11 | 266537 | 29 | 1817692 | 29 | 526249 |
|  |  | 6 | 7 | 60765 | 29 | 822014 | 19 | 158393 |
| (+)infected/ (-)treated | 8  Lung | 1 | 16 | 61330 | 31 | 453280 | 35 | 160707 |
|  |  | 2 | 8 | 59922 | 30 | 493065 | 19 | 95852 |
|  |  | 3 | 20 | 166847 | 33 | 931026 | 39 | 361718 |
|  |  | 4 | 12 | 47308 | 28 | 237085 | 29 | 67687 |
|  |  | 5 | 14 | 115554 | 32 | 839892 | 34 | 284015 |
|  |  | 6 | 16 | 87375 | 31 | 494777 | 42 | 208310 |

| Group | Day/ Organ | Mouse | Weight (g) | Resus. Vol. (ml) | Live count (cells/ ml) | Total # cells | Total # cells/g |
| --- | --- | --- | --- | --- | --- | --- | --- |
| (-)infected/ (+)treated | 10  Lung | 1 | 0.108 | 0.15 | 2730000 | 409500 | 3791667 |
|  |  | 2 | 0.065 | 0.15 | 2040000 | 306000 | 4707692 |
|  |  | 3 | 0.07 | 0.15 | 2600000 | 390000 | 5571429 |
|  |  | 4 | 0.064 | 0.15 | 2090000 | 313500 | 4898438 |
|  |  | 5 | 0.07 | 0.15 | 2050000 | 307500 | 4392857 |
|  |  | 6 | 0.072 | 0.15 | 943000 | 141450 | 1964583 |
| (+)infected/(+)treated | 10  Lung | 1 | 0.09 | 0.15 | 4920000 | 738000 | 8200000 |
|  |  | 2 | 0.112 | 0.15 | 3630000 | 544500 | 4861607 |
|  |  | 3 | 0.072 | 0.15 | 2500000 | 375000 | 5208333 |
|  |  | 4 | 0.097 | 0.15 | 8770000 | 1315500 | 13561856 |
|  |  | 5 | 0.083 | 0.15 | 4200000 | 630000 | 7590361 |
|  |  | 6 | 0.092 | 0.15 | 7800000 | 1170000 | 12717391 |
| (+)infected/(-)treated | 10  Lung | 1 | 0.102 | 0.15 | 6240000 | 936000 | 9176471 |
|  |  | 3 | 0.159 | 0.15 | 11800000 | 1770000 | 11132075 |

| Group | Day/ Organ | Mouse | Total events | Singlets | Lympho-  cytes | CD3 | CD4 | CD4 CD69 | CD8 | CD8 CD69 |
| --- | --- | --- | --- | --- | --- | --- | --- | --- | --- | --- |
| (-)infected/ (+)treated | 10  Lung | 1 | 78808 | 69289 | 42157 | 17809 | 12313 | 54 | 5038 | 33 |
|  |  | 2 | 44997 | 40198 | 25051 | 11244 | 7869 | 51 | 2898 | 43 |
|  |  | 3 | 70750 | 61749 | 38554 | 18403 | 13091 | 94 | 4842 | 46 |
|  |  | 4 | 48274 | 42203 | 24732 | 11950 | 8600 | 145 | 2977 | 35 |
|  |  | 5 | 56261 | 50010 | 31975 | 12283 | 8374 | 43 | 3354 | 42 |
|  |  | 6 | 20848 | 18547 | 11096 | 4925 | 3541 | 19 | 1255 | 5 |
| (+)infected/ (+)treated | 10  Lung | 1 | 139569 | 125133 | 87732 | 32844 | 19410 | 1605 | 10148 | 1872 |
|  |  | 2 | 87863 | 80499 | 55294 | 25714 | 17160 | 788 | 6773 | 950 |
|  |  | 3 | 66691 | 59857 | 43043 | 19642 | 13861 | 92 | 5128 | 49 |
|  |  | 4 | 208397 | 186984 | 135526 | 58131 | 34818 | 2991 | 17485 | 3994 |
|  |  | 5 | 97437 | 84579 | 51757 | 24779 | 16586 | 1183 | 6730 | 842 |
|  |  | 6 | 151305 | 133155 | 91737 | 46046 | 29573 | 2855 | 13366 | 3053 |
| (+)infected/ (-)treated | 10  Lung | 1 | 119239 | 107251 | 57947 | 28755 | 17467 | 2393 | 8389 | 3090 |
|  |  | 3 | 304103 | 271376 | 125185 | 38202 | 18981 | 3586 | 10923 | 4450 |

| Group | Day/ Organ | Mouse | %  Lymphocytes | Total # lymphoctyes/g | % CD3 | Total #CD3/g | % CD4 | Total # CD4/g |
| --- | --- | --- | --- | --- | --- | --- | --- | --- |
| (-)infected/ (+)treated | 10  Lung | 1 | 61 | 2306936 | 42 | 974553 | 69 | 673798 |
|  |  | 2 | 62 | 2933788 | 45 | 1316814 | 70 | 921559 |
|  |  | 3 | 62 | 3478613 | 48 | 1660448 | 71 | 1181162 |
|  |  | 4 | 59 | 2870605 | 48 | 1387018 | 72 | 998189 |
|  |  | 5 | 64 | 2808670 | 38 | 1078933 | 68 | 735569 |
|  |  | 6 | 60 | 1175339 | 44 | 521679 | 72 | 375079 |
| (+)infected/ (+)treated | 10  Lung | 1 | 70 | 5749102 | 37 | 2152276 | 59 | 1271943 |
|  |  | 2 | 69 | 3339392 | 47 | 1552956 | 67 | 1036350 |
|  |  | 3 | 72 | 3745298 | 46 | 1709108 | 71 | 1206086 |
|  |  | 4 | 72 | 9829633 | 43 | 4216212 | 60 | 2525332 |
|  |  | 5 | 61 | 4644821 | 48 | 2223738 | 67 | 1488475 |
|  |  | 6 | 69 | 8761634 | 50 | 4397770 | 64 | 2824463 |
| (+)infected/ (-)treated | 10  Lung | 1 | 54 | 4957986 | 50 | 2460298 | 61 | 1494489 |
|  |  | 3 | 46 | 5135196 | 31 | 1567079 | 50 | 778617 |

| Group | Day/ Organ | Mouse | % CD4  CD69 | Total # CD4CD69/g | % CD8 | Total # CD8/g | % CD8CD69 | Total # CD8CD69/g |
| --- | --- | --- | --- | --- | --- | --- | --- | --- |
| (-)infected/ (+)treated | 10  Lung | 1 | 0 | 2955 | 28 | 275692 | 1 | 1806 |
|  |  | 2 | 1 | 5973 | 26 | 339392 | 1 | 5036 |
|  |  | 3 | 1 | 8481 | 26 | 436879 | 1 | 4150 |
|  |  | 4 | 2 | 16830 | 25 | 345536 | 1 | 4062 |
|  |  | 5 | 1 | 3777 | 27 | 294614 | 1 | 3689 |
|  |  | 6 | 1 | 2013 | 25 | 132935 | 0 | 530 |
| (+)infected/ (+)treated | 10  Lung | 1 | 8 | 105176 | 31 | 665001 | 18 | 122673 |
|  |  | 2 | 5 | 47590 | 26 | 409044 | 14 | 57374 |
|  |  | 3 | 1 | 8005 | 26 | 446202 | 1 | 4264 |
|  |  | 4 | 9 | 216936 | 30 | 1268178 | 23 | 289683 |
|  |  | 5 | 7 | 106166 | 27 | 603969 | 13 | 75563 |
|  |  | 6 | 10 | 272676 | 29 | 1276562 | 23 | 291586 |
| (+)infected/ (-)treated | 10  Lung | 1 | 14 | 204747 | 29 | 717769 | 37 | 264383 |
|  |  | 3 | 19 | 147101 | 29 | 448071 | 41 | 182543 |

| Group | Day/ Organ | Mouse | Weight (g) | Resus. Vol. (ml) | Live count (cells/ ml) | Total # cells | Total # cells/g |
| --- | --- | --- | --- | --- | --- | --- | --- |
| Uninfected/untreated | 0  Spleen | 1 | 0.034 | 1 | 21100000 | 21100000 | 620588235 |
|  |  | 2 | 0.128 | 1 | 28200000 | 28200000 | 220312500 |
|  |  | 3 | 0.122 | 1 | 32400000 | 32400000 | 265573770 |
|  |  | 4 | 0.218 | 1 | 38100000 | 38100000 | 174770642 |

| Group | Day/ Organ | Mouse | Total events | Singlets | Lympho-cytes | CD3 | CD4 | CD4 CD69 | CD8 | CD8 CD69 |
| --- | --- | --- | --- | --- | --- | --- | --- | --- | --- | --- |
| Uninfected/ untreated | 0  Spleen | 1 | 251230 | 237491 | 220463 | 133195 | 82129 | 3213 | 47761 | 1774 |
|  |  | 2 | 235727 | 222510 | 205908 | 116355 | 71718 | 3121 | 41618 | 1620 |
|  |  | 3 | 217005 | 206103 | 185301 | 104572 | 65575 | 2662 | 36157 | 1323 |
|  |  | 4 | 215044 | 204544 | 188579 | 117590 | 74865 | 2720 | 39501 | 1431 |

| Group | Day/ Organ | Mouse | %  Lymphocytes | Total # lymphoctyes/g | % CD3 | Total # CD3/g | % CD4 | Total # CD4/g |
| --- | --- | --- | --- | --- | --- | --- | --- | --- |
| Uninfected/ untreated | 0  Spleen | 1 | 93 | 576092332 | 60 | 348052137 | 62 | 214611464 |
|  |  | 2 | 93 | 203874461 | 57 | 115205883 | 62 | 71009716 |
|  |  | 3 | 90 | 238769379 | 56 | 134746124 | 63 | 84496587 |
|  |  | 4 | 92 | 161129502 | 62 | 100473638 | 64 | 63967675 |

| Group | Day/ Organ | Mouse | % CD4  CD69 | Total # CD4CD69/g | % CD8 | Total # CD8/g | % CD8CD69 | Total # CD8CD69/g |
| --- | --- | --- | --- | --- | --- | --- | --- | --- |
| Uninfected/ untreated | 0  Spleen | 1 | 4 | 8395897 | 36 | 124804370 | 4 | 4635643 |
|  |  | 2 | 4 | 3090177 | 36 | 41206982 | 4 | 1604001 |
|  |  | 3 | 4 | 3430117 | 35 | 46590058 | 4 | 1704750 |
|  |  | 4 | 4 | 2324078 | 34 | 33751247 | 4 | 1222704 |

| Group | Day/ Organ | Mouse | Weight (g) | Resus. Vol. (ml) | Live count (cells/ ml) | Total # cells | Total # cells/g |
| --- | --- | --- | --- | --- | --- | --- | --- |
| (-)infected/ (+)treated | 2  Spleen | 1 | 0.058 | 1 | 24500000 | 24500000 | 422413793 |
|  |  | 2 | 0.04 | 1 | 16400000 | 16400000 | 410000000 |
|  |  | 3 | 0.07 | 1 | 24400000 | 24400000 | 348571429 |
|  |  | 4 | 0.066 | 1 | 18000000 | 18000000 | 272727273 |
|  |  | 5 | 0.147 | 1 | 9390000 | 9390000 | 63877551 |
|  |  | 6 | 0.201 | 1 | 19600000 | 19600000 | 97512438 |
| (+)infected/(+)treated | 2  Spleen | 1 | 0.078 | 1 | 14000000 | 14000000 | 179487179 |
|  |  | 2 | 0.156 | 1 | 20700000 | 20700000 | 132692308 |
|  |  | 3 | 0.19 | 1 | 23900000 | 23900000 | 125789474 |
|  |  | 4 | 0.068 | 1 | 18600000 | 18600000 | 273529412 |
|  |  | 5 | 0.162 | 1 | 24200000 | 24200000 | 149382716 |
|  |  | 6 | 0.139 | 1 | 21600000 | 21600000 | 155395683 |
| (+)infected/(-)treated | 2  Spleen | 1 | 0.195 | 1 | 21500000 | 21500000 | 110256410 |
|  |  | 2 | 0.033 | 1 | 23700000 | 23700000 | 718181818 |
|  |  | 3 | 0.202 | 1 | 28300000 | 28300000 | 140099010 |
|  |  | 4 | 0.077 | 1 | 36100000 | 36100000 | 468831169 |
|  |  | 5 | 0.156 | 1 | 30100000 | 30100000 | 192948718 |
|  |  | 6 | 0.144 | 1 | 23700000 | 23700000 | 164583333 |

| Group | Day/ Organ | Mouse | Total events | Singlets | Lympho-  cytes | CD3 | CD4 | CD4 CD69 | CD8 | CD8 CD69 |
| --- | --- | --- | --- | --- | --- | --- | --- | --- | --- | --- |
| (-)infected/ (+)treated | 2  Spleen | 1 | 227513 | 220547 | 179707 | 94236 | 58220 | 2876 | 30175 | 1412 |
|  |  | 2 | 202813 | 196744 | 152297 | 92929 | 59688 | 2234 | 29055 | 992 |
|  |  | 3 | 215451 | 208164 | 168767 | 93056 | 59498 | 3660 | 29503 | 1748 |
|  |  | 4 | 241277 | 231710 | 195145 | 97757 | 60987 | 3308 | 32301 | 1756 |
|  |  | 5 | 238623 | 230464 | 188592 | 103085 | 62066 | 3183 | 36640 | 1710 |
|  |  | 6 | 232185 | 223166 | 190811 | 104623 | 65480 | 4010 | 34745 | 2021 |
| (+)infected/ (+)treated | 2  Spleen | 1 | 97271 | 94537 | 74411 | 43140 | 27042 | 1399 | 14198 | 700 |
|  |  | 2 | 230695 | 223315 | 182712 | 106485 | 65921 | 3118 | 35748 | 1615 |
|  |  | 3 | 97145 | 94976 | 76969 | 48561 | 30765 | 1319 | 16066 | 686 |
|  |  | 4 | 273541 | 263597 | 226063 | 123901 | 75426 | 4007 | 42788 | 2031 |
|  |  | 5 | 308913 | 295090 | 246099 | 131416 | 81497 | 4681 | 43542 | 2228 |
|  |  | 6 | 223739 | 215653 | 176886 | 97139 | 60288 | 3339 | 33050 | 1635 |
| (+)infected/ (-)treated | 2  Spleen | 1 | 123247 | 121111 | 94352 | 63867 | 40906 | 1662 | 20544 | 743 |
|  |  | 2 | 160580 | 157214 | 127728 | 86607 | 56052 | 1970 | 27407 | 901 |
|  |  | 3 | 157657 | 154172 | 128848 | 84821 | 53042 | 1952 | 28908 | 954 |
|  |  | 4 | 124897 | 121518 | 103707 | 63887 | 41853 | 1591 | 19755 | 633 |
|  |  | 5 | 128168 | 125507 | 101698 | 40542 | 32120 | 664 | 6425 | 129 |
|  |  | 6 | 119246 | 116132 | 100073 | 63070 | 39394 | 1681 | 21559 | 788 |

| Group | Day/ Organ | Mouse | %  Lymphocytes | Total # lymphoctyes/g | % CD3 | Total #CD3/g | % CD4 | Total # CD4/g |
| --- | --- | --- | --- | --- | --- | --- | --- | --- |
| (-)infected/ (+)treated | 2  Spleen | 1 | 81 | 344192918 | 52 | 180490264 | 62 | 111508799 |
|  |  | 2 | 77 | 317375727 | 61 | 193657189 | 64 | 124385394 |
|  |  | 3 | 81 | 282600999 | 55 | 155822634 | 64 | 99629633 |
|  |  | 4 | 84 | 229689541 | 50 | 115061931 | 62 | 71782910 |
|  |  | 5 | 82 | 52271917 | 55 | 28572000 | 60 | 17202791 |
|  |  | 6 | 86 | 83374913 | 55 | 45715045 | 63 | 28611502 |
| (+)infected/ (+)treated | 2  Spleen | 1 | 79 | 141276120 | 58 | 81905253 | 63 | 51341721 |
|  |  | 2 | 82 | 108566272 | 58 | 63272688 | 62 | 39169826 |
|  |  | 3 | 81 | 101940385 | 63 | 64315855 | 63 | 40746222 |
|  |  | 4 | 86 | 234581120 | 55 | 128569626 | 61 | 78268074 |
|  |  | 5 | 83 | 124582117 | 53 | 66526412 | 62 | 41256034 |
|  |  | 6 | 82 | 127460879 | 55 | 69996621 | 62 | 43442451 |
| (+)infected/ (-)treated | 2  Spleen | 1 | 78 | 85895689 | 68 | 58142911 | 64 | 37239794 |
|  |  | 2 | 81 | 583484469 | 68 | 395636347 | 65 | 256055614 |
|  |  | 3 | 84 | 117086613 | 66 | 77078446 | 63 | 48200268 |
|  |  | 4 | 85 | 400114173 | 62 | 246483787 | 66 | 161473945 |
|  |  | 5 | 81 | 156345851 | 40 | 62327415 | 79 | 49379818 |
|  |  | 6 | 86 | 141824372 | 63 | 89383381 | 62 | 55829537 |

| Group | Day/ Organ | Mouse | % CD4  CD69 | Total # CD4CD69/g | % CD8 | Total # CD8/g | % CD8CD69 | Total # CD8CD69/g |
| --- | --- | --- | --- | --- | --- | --- | --- | --- |
| (-)infected/ (+)treated | 2  Spleen | 1 | 5 | 5508404 | 32 | 57794194 | 5 | 2704404 |
|  |  | 2 | 4 | 4655491 | 31 | 60548479 | 3 | 2067255 |
|  |  | 3 | 6 | 6128684 | 32 | 49402888 | 6 | 2927033 |
|  |  | 4 | 5 | 3893582 | 33 | 38018919 | 5 | 2066847 |
|  |  | 5 | 5 | 882230 | 36 | 10155484 | 5 | 473960 |
|  |  | 6 | 6 | 1752170 | 33 | 15181836 | 6 | 883076 |
| (+)infected/ (+)treated | 2  Spleen | 1 | 5 | 2656130 | 33 | 26956207 | 5 | 1329014 |
|  |  | 2 | 5 | 1852695 | 34 | 21241227 | 5 | 959622 |
|  |  | 3 | 4 | 1746929 | 33 | 21278362 | 4 | 908562 |
|  |  | 4 | 5 | 4157985 | 35 | 44400264 | 5 | 2107529 |
|  |  | 5 | 6 | 2369652 | 33 | 22042164 | 5 | 1127875 |
|  |  | 6 | 6 | 2406024 | 34 | 23815237 | 5 | 1178152 |
| (+)infected/ (-)treated | 2  Spleen | 1 | 4 | 1513043 | 32 | 18702741 | 4 | 676409 |
|  |  | 2 | 4 | 8999314 | 32 | 125200104 | 3 | 4115930 |
|  |  | 3 | 4 | 1773819 | 34 | 26269246 | 3 | 866918 |
|  |  | 4 | 4 | 6138271 | 31 | 76217184 | 3 | 2442191 |
|  |  | 5 | 2 | 1020803 | 16 | 9877501 | 2 | 198319 |
|  |  | 6 | 4 | 2382329 | 34 | 30553612 | 4 | 1116761 |

| Group | Day/ Organ | Mouse | Weight (g) | Resus. Vol. (ml) | Live count (cells/ ml) | Total # cells | Total # cells/g |
| --- | --- | --- | --- | --- | --- | --- | --- |
| (-)infected/ (+)treated | 4  Spleen | 1 | 0.06 | 1 | 34900000 | 34900000 | 581666667 |
|  |  | 2 | 0.039 | 1 | 20100000 | 20100000 | 515384615 |
|  |  | 3 | 0.038 | 1 | 35900000 | 35900000 | 944736842 |
|  |  | 4 | 0.04 | 1 | 53500000 | 53500000 | 1337500000 |
|  |  | 5 | - | 1 | 26600000 | 26600000 |  |
|  |  | 6 | 0.049 | 1 | 42100000 | 42100000 | 859183673 |
| (+)infected/(+)treated | 4  Spleen | 1 | 0.059 | 1 | 53900000 | 53900000 | 913559322 |
|  |  | 2 | 0.054 | 1 | 40100000 | 40100000 | 742592593 |
|  |  | 3 | 0.047 | 1 | 39500000 | 39500000 | 840425532 |
|  |  | 4 | 0.04 | 1 | 36700000 | 36700000 | 917500000 |
|  |  | 5 | 0.044 | 1 | 38100000 | 38100000 | 865909091 |
|  |  | 6 | 0.032 | 1 | 46100000 | 46100000 | 1440625000 |
| (+)infected/(-)treated | 4  Spleen | 1 | 0.052 | 1 | 35000000 | 35000000 | 673076923 |
|  |  | 2 | 0.065 | 1 | 67500000 | 67500000 | 1038461538 |
|  |  | 3 | 0.056 | 1 | 42000000 | 42000000 | 750000000 |
|  |  | 4 | 0.067 | 1 | 32100000 | 32100000 | 479104478 |
|  |  | 5 | 0.056 | 1 | 45000000 | 45000000 | 803571429 |
|  |  | 6 | 0.068 | 1 | 38700000 | 38700000 | 569117647 |

| Group | Day/ Organ | Mouse | Total events | Singlets | Lympho-  cytes | CD3 | CD4 | CD4 CD69 | CD8 | CD8 CD69 |
| --- | --- | --- | --- | --- | --- | --- | --- | --- | --- | --- |
| (-)infected/ (+)treated | 4  Spleen | 1 | 231867 | 225004 | 178083 | 95916 | 61767 | 2569 | 31118 | 1038 |
|  |  | 2 | 192826 | 188378 | 150210 | 93664 | 58369 | 2170 | 32527 | 1106 |
|  |  | 3 | 267066 | 258929 | 209106 | 112566 | 72377 | 3337 | 36587 | 1392 |
|  |  | 4 | 216959 | 211157 | 166732 | 100415 | 62673 | 3060 | 34087 | 1244 |
|  |  | 5 | 138576 | 135405 | 107955 | 62889 | 39038 | 1774 | 21661 | 790 |
|  |  | 6 | 160874 | 156516 | 131298 | 73893 | 45817 | 2112 | 25210 | 959 |
| (+)infected/ (+)treated | 4  Spleen | 1 | 68570 | 66593 | 52918 | 31911 | 19828 | 1702 | 10848 | 1546 |
|  |  | 2 | 180680 | 173098 | 144679 | 86439 | 50725 | 4701 | 32243 | 5443 |
|  |  | 3 | 168706 | 162623 | 136988 | 80900 | 49887 | 2410 | 28701 | 1550 |
|  |  | 4 | 363484 | 339526 | 290687 | 157326 | 91375 | 10174 | 59891 | 9101 |
|  |  | 5 | 157990 | 152941 | 128804 | 79107 | 49007 | 2668 | 27540 | 1826 |
|  |  | 6 | 154759 | 148110 | 123411 | 73006 | 43513 | 4001 | 26960 | 3209 |
| (+)infected/ (-)treated | 4  Spleen | 1 | 111080 | 105568 | 84875 | 45638 | 27447 | 4223 | 16254 | 4083 |
|  |  | 2 | 70726 | 67777 | 56657 | 34524 | 21166 | 4881 | 11744 | 3879 |
|  |  | 3 | 143536 | 136449 | 112774 | 64248 | 40337 | 7482 | 21226 | 6889 |
|  |  | 4 | 298509 | 282869 | 241174 | 138763 | 83137 | 17248 | 49555 | 16107 |
|  |  | 5 | 129117 | 121808 | 100982 | 53935 | 33048 | 4220 | 18777 | 4364 |
|  |  | 6 | 238153 | 227269 | 197521 | 120282 | 72341 | 8162 | 44209 | 7883 |

| Group | Day/ Organ | Mouse | %  Lymphocytes | Total # lymphoctyes/g | % CD3 | Total #CD3/g | % CD4 | Total # CD4/g |
| --- | --- | --- | --- | --- | --- | --- | --- | --- |
| (-)infected/ (+)treated | 4  Spleen | 1 | 79 | 460369349 | 54 | 247956214 | 64 | 159676295 |
|  |  | 2 | 80 | 410960532 | 62 | 256255957 | 62 | 159692133 |
|  |  | 3 | 81 | 762951010 | 54 | 410712000 | 64 | 264077096 |
|  |  | 4 | 79 | 1056105410 | 60 | 636043619 | 62 | 396980150 |
|  |  | 5 | 80 |  | 58 |  | 62 |  |
|  |  | 6 | 84 | 720751220 | 56 | 405630473 | 62 | 251509228 |
| (+)infected/ (+)treated | 4  Spleen | 1 | 79 | 725958167 | 60 | 437772612 | 62 | 272011386 |
|  |  | 2 | 84 | 620674726 | 60 | 370824395 | 59 | 217610887 |
|  |  | 3 | 84 | 707945449 | 59 | 418086160 | 62 | 257812908 |
|  |  | 4 | 86 | 785522530 | 54 | 425141536 | 58 | 246922364 |
|  |  | 5 | 84 | 729252160 | 61 | 447881670 | 62 | 277463903 |
|  |  | 6 | 83 | 1200384659 | 59 | 710109167 | 60 | 423238914 |
| (+)infected/ (-)treated | 4  Spleen | 1 | 80 | 541143186 | 54 | 290977234 | 60 | 174995664 |
|  |  | 2 | 84 | 868083795 | 61 | 528967735 | 61 | 324299938 |
|  |  | 3 | 83 | 619868962 | 57 | 353142933 | 63 | 221714707 |
|  |  | 4 | 85 | 408484292 | 58 | 235027432 | 60 | 140811856 |
|  |  | 5 | 83 | 666181614 | 53 | 355810989 | 61 | 218018756 |
|  |  | 6 | 87 | 494623934 | 61 | 301205219 | 60 | 181153346 |

| Group | Day/ Organ | Mouse | % CD4  CD69 | Total # CD4CD69/g | % CD8 | Total # CD8/g | % CD8CD69 | Total # CD8CD69/g |
| --- | --- | --- | --- | --- | --- | --- | --- | --- |
| (-)infected/ (+)treated | 4  Spleen | 1 | 4 | 6641223 | 32 | 80444362 | 3 | 2683375 |
|  |  | 2 | 4 | 5936917 | 35 | 88990834 | 3 | 3025913 |
|  |  | 3 | 5 | 12175488 | 33 | 133492528 | 4 | 5078897 |
|  |  | 4 | 5 | 19382497 | 34 | 215912153 | 4 | 7879682 |
|  |  | 5 | 5 |  | 34 |  | 4 |  |
|  |  | 6 | 5 | 11593677 | 34 | 138388538 | 4 | 5264364 |
| (+)infected/ (+)treated | 4  Spleen | 1 | 9 | 23348970 | 34 | 148818818 | 14 | 21208876 |
|  |  | 2 | 9 | 20167349 | 37 | 138322875 | 17 | 23350538 |
|  |  | 3 | 5 | 12454730 | 35 | 148324980 | 5 | 8010303 |
|  |  | 4 | 11 | 27493167 | 38 | 161843254 | 15 | 24593603 |
|  |  | 5 | 5 | 15105468 | 35 | 155923764 | 7 | 10338300 |
|  |  | 6 | 9 | 38916620 | 37 | 262232462 | 12 | 31213055 |
| (+)infected/ (-)treated | 4  Spleen | 1 | 15 | 26924862 | 36 | 103631709 | 25 | 26032255 |
|  |  | 2 | 23 | 74785411 | 34 | 179938509 | 33 | 59433028 |
|  |  | 3 | 19 | 41125256 | 33 | 116669965 | 32 | 37865796 |
|  |  | 4 | 21 | 29213502 | 36 | 83932924 | 33 | 27280953 |
|  |  | 5 | 13 | 27839480 | 35 | 123872494 | 23 | 28789453 |
|  |  | 6 | 11 | 20438943 | 37 | 110706353 | 18 | 19740283 |

| Group | Day/ Organ | Mouse | Weight (g) | Resus. Vol. (ml) | Live count (cells/ ml) | Total # cells | Total # cells/g |
| --- | --- | --- | --- | --- | --- | --- | --- |
| (-)infected/ (+)treated | 6  Spleen | 1 | 0.049 | 1 | 31600000 | 31600000 | 644897959 |
|  |  | 2 | 0.044 | 1 | 42500000 | 42500000 | 965909091 |
|  |  | 3 | 0.047 | 1 | 47700000 | 47700000 | 1014893617 |
|  |  | 4 | 0.056 | 1 | 45500000 | 45500000 | 812500000 |
|  |  | 5 | 0.054 | 1 | 46500000 | 46500000 | 861111111 |
|  |  | 6 | 0.037 | 1 | 39700000 | 39700000 | 1072972973 |
| (+)infected/(+)treated | 6  Spleen | 1 | 0.046 | 1 | 42400000 | 42400000 | 921739130 |
|  |  | 2 | 0.046 | 1 | 32500000 | 32500000 | 706521739 |
|  |  | 3 | 0.074 | 1 | 57900000 | 57900000 | 782432432 |
|  |  | 4 | 0.077 | 1 | 52700000 | 52700000 | 684415584 |
|  |  | 5 | - | 1 | 34100000 | 34100000 |  |
|  |  | 6 | 0.058 | 1 | 38700000 | 38700000 | 667241379 |
| (+)infected/(-)treated | 6  Spleen | 1 | 0.077 | 1 | 52000000 | 52000000 | 675324675 |
|  |  | 2 | 0.057 | 1 | 42800000 | 42800000 | 750877193 |
|  |  | 3 | 0.052 | 1 | 31100000 | 31100000 | 598076923 |
|  |  | 4 | 0.063 | 1 | 48700000 | 48700000 | 773015873 |
|  |  | 5 | 0.082 | 1 | 71000000 | 71000000 | 865853659 |
|  |  | 6 | 0.057 | 1 | 37600000 | 37600000 | 659649123 |

| Group | Day/ Organ | Mouse | Total events | Singlets | Lympho-  cytes | CD3 | CD4 | CD4 CD69 | CD8 | CD8 CD69 |
| --- | --- | --- | --- | --- | --- | --- | --- | --- | --- | --- |
| (-)infected/ (+)treated | 6  Spleen | 1 | 329578 | 318147 | 250330 | 146711 | 94580 | 4703 | 47584 | 1943 |
|  |  | 2 | 234789 | 229035 | 177877 | 113462 | 73523 | 3234 | 36750 | 1276 |
|  |  | 3 | 236675 | 228975 | 177126 | 108414 | 71827 | 2816 | 33384 | 1168 |
|  |  | 4 | 241903 | 234577 | 185027 | 109844 | 71722 | 3204 | 34492 | 1356 |
|  |  | 5 | 217754 | 210584 | 179767 | 109119 | 71290 | 2848 | 34617 | 1215 |
|  |  | 6 | 269933 | 261022 | 212396 | 132744 | 87947 | 3437 | 41401 | 1340 |
| (+)infected/ (+)treated | 6  Spleen | 1 | 300625 | 285934 | 237867 | 142935 | 87379 | 6776 | 51464 | 3203 |
|  |  | 2 | 262196 | 253336 | 209707 | 106563 | 66066 | 3497 | 37026 | 1601 |
|  |  | 3 | 280571 | 267622 | 229561 | 136858 | 86428 | 5285 | 46001 | 2448 |
|  |  | 4 | 295186 | 282925 | 235968 | 124617 | 78473 | 5315 | 42185 | 2375 |
|  |  | 5 | 324000 | 310262 | 267761 | 158454 | 102276 | 5725 | 51529 | 2700 |
|  |  | 6 | 246426 | 238372 | 198996 | 119575 | 75538 | 5062 | 40272 | 2496 |
| (+)infected/ (-)treated | 6  Spleen | 1 | 218239 | 206598 | 182057 | 90034 | 50160 | 6350 | 35609 | 6043 |
|  |  | 2 | 224745 | 216082 | 186156 | 114838 | 66218 | 8819 | 44057 | 9189 |
|  |  | 3 | 343767 | 326916 | 292252 | 176776 | 111237 | 7131 | 60277 | 5633 |
|  |  | 4 | 303575 | 286621 | 249039 | 135989 | 80194 | 9394 | 50630 | 8042 |
|  |  | 5 | 235249 | 224676 | 190177 | 104305 | 57000 | 7186 | 42354 | 8323 |
|  |  | 6 | 191091 | 181809 | 152430 | 76697 | 44193 | 4721 | 28948 | 4394 |

| Group | Day/ Organ | Mouse | %  Lymphocytes | Total # lymphoctyes/g | % CD3 | Total #CD3/g | % CD4 | Total # CD4/g |
| --- | --- | --- | --- | --- | --- | --- | --- | --- |
| (-)infected/ (+)treated | 6  Spleen | 1 | 79 | 507429918 | 59 | 297389648 | 64 | 191717819 |
|  |  | 2 | 78 | 750160505 | 64 | 478503186 | 65 | 310068479 |
|  |  | 3 | 77 | 785081545 | 61 | 480527030 | 66 | 318361235 |
|  |  | 4 | 79 | 640874585 | 59 | 380464624 | 65 | 248422160 |
|  |  | 5 | 85 | 735095549 | 61 | 446204761 | 65 | 291516027 |
|  |  | 6 | 81 | 873087968 | 62 | 545665593 | 66 | 361520309 |
| (+)infected/ (+)treated | 6  Spleen | 1 | 83 | 766789965 | 60 | 460766410 | 61 | 281675644 |
|  |  | 2 | 83 | 584846032 | 51 | 297190593 | 62 | 184249634 |
|  |  | 3 | 86 | 671155479 | 60 | 400124571 | 63 | 252685019 |
|  |  | 4 | 83 | 570823280 | 53 | 301457336 | 63 | 189831737 |
|  |  | 5 | 86 |  | 59 |  | 65 |  |
|  |  | 6 | 83 | 557021653 | 60 | 334709563 | 63 | 211442952 |
| (+)infected/ (-)treated | 6  Spleen | 1 | 88 | 595105395 | 49 | 294301890 | 56 | 163962312 |
|  |  | 2 | 86 | 646885417 | 62 | 399057927 | 58 | 230105173 |
|  |  | 3 | 89 | 534660821 | 60 | 323403095 | 63 | 203502682 |
|  |  | 4 | 87 | 671657345 | 55 | 366761876 | 59 | 216282948 |
|  |  | 5 | 85 | 732901828 | 55 | 401969351 | 55 | 219665912 |
|  |  | 6 | 84 | 553054666 | 50 | 278276151 | 58 | 160343403 |

| Group | Day/ Organ | Mouse | % CD4  CD69 | Total # CD4CD69/g | % CD8 | Total # CD8/g | % CD8CD69 | Total # CD8CD69/g |
| --- | --- | --- | --- | --- | --- | --- | --- | --- |
| (-)infected/ (+)treated | 6  Spleen | 1 | 5 | 9533188 | 32 | 96454860 | 4 | 3938546 |
|  |  | 2 | 4 | 13638745 | 32 | 154985741 | 3 | 5381274 |
|  |  | 3 | 4 | 12481452 | 31 | 147969029 | 3 | 5176966 |
|  |  | 4 | 4 | 11097635 | 31 | 119469300 | 4 | 4696752 |
|  |  | 5 | 4 | 11645920 | 32 | 141554360 | 4 | 4968326 |
|  |  | 6 | 4 | 14128342 | 31 | 170185479 | 3 | 5508286 |
| (+)infected/ (+)treated | 6  Spleen | 1 | 8 | 21843168 | 36 | 165899762 | 6 | 10325216 |
|  |  | 2 | 5 | 9752686 | 35 | 103260784 | 4 | 4464984 |
|  |  | 3 | 6 | 15451478 | 34 | 134490716 | 5 | 7157089 |
|  |  | 4 | 7 | 12857361 | 34 | 102048498 | 6 | 5745293 |
|  |  | 5 | 6 |  | 33 |  | 5 |  |
|  |  | 6 | 7 | 14169348 | 34 | 112727774 | 6 | 6986703 |
| (+)infected/ (-)treated | 6  Spleen | 1 | 13 | 20756792 | 40 | 116398205 | 17 | 19753275 |
|  |  | 2 | 13 | 30645708 | 38 | 153096493 | 21 | 31931445 |
|  |  | 3 | 6 | 13045818 | 34 | 110273840 | 9 | 10305300 |
|  |  | 4 | 12 | 25335586 | 37 | 136548940 | 16 | 21689247 |
|  |  | 5 | 13 | 27693320 | 41 | 163223334 | 20 | 32075077 |
|  |  | 6 | 11 | 17128984 | 38 | 105030679 | 15 | 15942545 |

| Group | Day/ Organ | Mouse | Weight (g) | Resus. Vol. (ml) | Live count (cells/ ml) | Total # cells | Total # cells/g |
| --- | --- | --- | --- | --- | --- | --- | --- |
| (-)infected/ (+)treated | 8  Spleen | 1 | 0.054 | 1 | 52500000 | 52500000 | 972222222 |
|  |  | 2 | 0.039 | 1 | 42600000 | 42600000 | 1092307692 |
|  |  | 3 | 0.072 | 1 | 39500000 | 39500000 | 548611111 |
|  |  | 4 | 0.039 | 1 | 12500000 | 12500000 | 320512821 |
|  |  | 5 | 0.035 | 1 | 25200000 | 25200000 | 720000000 |
|  |  | 6 | 0.045 | 1 | 41900000 | 41900000 | 931111111 |
| (+)infected/(+)treated | 8  Spleen | 1 | 0.091 | 1 | 64100000 | 64100000 | 704395604 |
|  |  | 2 | 0.061 | 1 | 30100000 | 30100000 | 493442623 |
|  |  | 3 | 0.075 | 1 | 43700000 | 43700000 | 582666667 |
|  |  | 4 | 0.067 | 1 | 39200000 | 39200000 | 585074627 |
|  |  | 5 | 0.072 | 1 | 62700000 | 62700000 | 870833333 |
|  |  | 6 | 0.069 | 1 | 66500000 | 66500000 | 963768116 |
| (+)infected/(-)treated | 8  Spleen | 1 | 0.081 | 1 | 49200000 | 49200000 | 607407407 |
|  |  | 2 | 0.093 | 1 | 61500000 | 61500000 | 661290323 |
|  |  | 3 | 0.087 | 1 | 42100000 | 42100000 | 483908046 |
|  |  | 4 | 0.062 | 1 | 49700000 | 49700000 | 801612903 |
|  |  | 5 | 0.072 | 1 | 42600000 | 42600000 | 591666667 |
|  |  | 6 | 0.075 | 1 | 38300000 | 38300000 | 510666667 |

| Group | Day/ Organ | Mouse | Total events | Singlets | Lympho-cytes | CD3 | CD4 | CD4 CD69 | CD8 | CD8 CD69 |
| --- | --- | --- | --- | --- | --- | --- | --- | --- | --- | --- |
| (-)infected/ (+)treated | 8  Spleen | 1 | 228152 | 220455 | 180381 | 108893 | 71870 | 3241 | 34015 | 1261 |
|  |  | 2 | 136171 | 131830 | 111271 | 64288 | 42026 | 1444 | 20589 | 621 |
|  |  | 3 | 274836 | 262959 | 217165 | 121724 | 77635 | 3271 | 40233 | 1528 |
|  |  | 4 | 161677 | 156478 | 130277 | 81341 | 53745 | 2034 | 25634 | 927 |
|  |  | 5 | 229064 | 216146 | 184806 | 113523 | 73558 | 2696 | 37435 | 1210 |
|  |  | 6 | 250203 | 240245 | 202119 | 115285 | 72617 | 2881 | 39468 | 1477 |
| (+)infected/ (+)treated | 8  Spleen | 1 | 361971 | 344830 | 289241 | 133313 | 82964 | 5523 | 45070 | 2474 |
|  |  | 2 | 438000 | 418000 | 352866 | 219642 | 140426 | 9251 | 72362 | 3645 |
|  |  | 3 | 360824 | 346041 | 294474 | 152995 | 100871 | 5638 | 46712 | 2252 |
|  |  | 4 | 409000 | 392355 | 326262 | 165075 | 108154 | 5635 | 51745 | 2217 |
|  |  | 5 | 378584 | 364195 | 308307 | 159656 | 103348 | 6505 | 51169 | 2067 |
|  |  | 6 | 289710 | 278890 | 240543 | 123834 | 77360 | 4313 | 42437 | 1879 |
| (+)infected/ (-)treated | 8  Spleen | 1 | 459000 | 435000 | 349108 | 179858 | 114352 | 11457 | 57312 | 4342 |
|  |  | 2 | 415000 | 393880 | 332483 | 166106 | 103014 | 11453 | 57043 | 5112 |
|  |  | 3 | 468000 | 440000 | 347372 | 173293 | 104727 | 10993 | 58424 | 4567 |
|  |  | 4 | 358641 | 338815 | 288349 | 149468 | 92176 | 11845 | 51636 | 5088 |
|  |  | 5 | 326229 | 306700 | 258100 | 142450 | 86679 | 9505 | 50767 | 4079 |
|  |  | 6 | 435000 | 412000 | 341908 | 190353 | 120833 | 13169 | 61491 | 5558 |

| Group | Day/ Organ | Mouse | %  Lymphocytes | Total # lymphoctyes/g | % CD3 | Total #CD3/g | % CD4 | Total # CD4/g |
| --- | --- | --- | --- | --- | --- | --- | --- | --- |
| (-)infected/ (+)treated | 8  Spleen | 1 | 82 | 795493033 | 60 | 480225871 | 66 | 316951809 |
|  |  | 2 | 84 | 921961384 | 58 | 532672965 | 65 | 348216059 |
|  |  | 3 | 83 | 453071133 | 56 | 253952665 | 64 | 161969827 |
|  |  | 4 | 83 | 266845491 | 62 | 166610216 | 66 | 110085517 |
|  |  | 5 | 86 | 615603897 | 61 | 378154396 | 65 | 245027713 |
|  |  | 6 | 84 | 783347194 | 57 | 446806986 | 63 | 281439762 |
| (+)infected/ (+)treated | 8  Spleen | 1 | 84 | 590842122 | 46 | 272322858 | 62 | 169473297 |
|  |  | 2 | 84 | 416552930 | 62 | 259284030 | 64 | 165770751 |
|  |  | 3 | 85 | 495837730 | 52 | 257614233 | 66 | 169847415 |
|  |  | 4 | 83 | 486517613 | 51 | 246157674 | 66 | 161277825 |
|  |  | 5 | 85 | 737198513 | 52 | 381756385 | 65 | 247117295 |
|  |  | 6 | 86 | 831251296 | 51 | 427936681 | 62 | 267335155 |
| (+)infected/ (-)treated | 8  Spleen | 1 | 80 | 487473069 | 52 | 251142716 | 64 | 159674142 |
|  |  | 2 | 84 | 558210090 | 50 | 278877552 | 62 | 172951562 |
|  |  | 3 | 79 | 382036604 | 50 | 190586084 | 60 | 115177813 |
|  |  | 4 | 85 | 682213831 | 52 | 353630971 | 62 | 218082054 |
|  |  | 5 | 84 | 497910553 | 55 | 274805728 | 61 | 167215765 |
|  |  | 6 | 83 | 423788880 | 56 | 235939155 | 63 | 149770353 |

| Group | Day/ Organ | Mouse | % CD4  CD69 | Total # CD4CD69/g | % CD8 | Total # CD8/g | % CD8CD69 | Total # CD8CD69/g |
| --- | --- | --- | --- | --- | --- | --- | --- | --- |
| (-)infected/ (+)treated | 8  Spleen | 1 | 5 | 14293040 | 31 | 150008568 | 4 | 5561100 |
|  |  | 2 | 3 | 11964593 | 32 | 170594880 | 3 | 5145438 |
|  |  | 3 | 4 | 6824284 | 33 | 83938070 | 4 | 3187865 |
|  |  | 4 | 4 | 4166228 | 32 | 52505947 | 4 | 1898768 |
|  |  | 5 | 4 | 8980596 | 33 | 124699046 | 3 | 4030609 |
|  |  | 6 | 4 | 11165815 | 34 | 152965070 | 4 | 5724369 |
| (+)infected/ (+)treated | 8  Spleen | 1 | 7 | 11282014 | 34 | 92065974 | 5 | 5053721 |
|  |  | 2 | 7 | 10920664 | 33 | 85422237 | 5 | 4302867 |
|  |  | 3 | 6 | 9493311 | 31 | 78654048 | 5 | 3791936 |
|  |  | 4 | 5 | 8402838 | 31 | 77161465 | 4 | 3305961 |
|  |  | 5 | 6 | 15554225 | 32 | 122351133 | 4 | 4942442 |
|  |  | 6 | 6 | 14904557 | 34 | 146650750 | 4 | 6493314 |
| (+)infected/ (-)treated | 8  Spleen | 1 | 10 | 15997854 | 32 | 80026973 | 8 | 6062903 |
|  |  | 2 | 11 | 19228593 | 34 | 95770244 | 9 | 8582604 |
|  |  | 3 | 10 | 12090003 | 34 | 64254190 | 8 | 5022746 |
|  |  | 4 | 13 | 28024452 | 35 | 122167212 | 10 | 12037857 |
|  |  | 5 | 11 | 18336458 | 36 | 97936556 | 8 | 7868954 |
|  |  | 6 | 11 | 16322741 | 32 | 76217000 | 9 | 6889042 |

| Group | Day/ Organ | Mouse | Weight (g) | Resus. Vol. (ml) | Live count (cells/ ml) | Total # cells | Total # cells/g |
| --- | --- | --- | --- | --- | --- | --- | --- |
| (-)infected/ (+)treated | 10  Spleen | 1 | 0.04 | 1 | 29300000 | 29300000 | 732500000 |
|  |  | 2 | 0.044 | 1 | 36500000 | 36500000 | 829545455 |
|  |  | 3 | 0.044 | 1 | 34700000 | 34700000 | 788636364 |
|  |  | 4 | 0.038 | 1 | 30800000 | 30800000 | 810526316 |
|  |  | 5 | 0.041 | 1 | 16400000 | 16400000 | 400000000 |
|  |  | 6 | 0.049 | 1 | 36700000 | 36700000 | 748979592 |
| (+)infected/(+)treated | 10  Spleen | 1 | 0.066 | 1 | 53600000 | 53600000 | 812121212 |
|  |  | 2 | 0.086 | 1 | 69700000 | 69700000 | 810465116 |
|  |  | 3 | 0.046 | 1 | 32700000 | 32700000 | 710869565 |
|  |  | 4 | 0.071 | 1 | 60900000 | 60900000 | 857746479 |
|  |  | 5 | 0.046 | 1 | 32300000 | 32300000 | 702173913 |
|  |  | 6 | 0.082 | 1 | 60400000 | 60400000 | 736585366 |
| (+)infected/(-)treated | 10  Spleen | 1 | 0.05 | 1 | 25300000 | 25300000 | 506000000 |
|  |  | 3 | 0.058 | 1 | 25800000 | 25800000 | 444827586 |

| Group | Day/ Organ | Mouse | Total events | Singlets | Lympho-  cytes | CD3 | CD4 | CD4 CD69 | CD8 | CD8 CD69 |
| --- | --- | --- | --- | --- | --- | --- | --- | --- | --- | --- |
| (-)infected/ (+)treated | 10  Spleen | 1 | 269064 | 260457 | 220419 | 117075 | 75401 | 2945 | 38564 | 1291 |
|  |  | 2 | 229067 | 222429 | 182763 | 117115 | 76237 | 3197 | 38132 | 1339 |
|  |  | 3 | 343656 | 330979 | 277272 | 160410 | 103327 | 4113 | 53451 | 1758 |
|  |  | 4 | 284715 | 273556 | 215570 | 123770 | 80708 | 3510 | 39968 | 1350 |
|  |  | 5 | 366673 | 353659 | 283777 | 161865 | 106159 | 4703 | 51625 | 2068 |
|  |  | 6 | 341843 | 329421 | 261114 | 142132 | 91534 | 3749 | 47160 | 1693 |
| (+)infected/ (+)treated | 10  Spleen | 1 | 331039 | 317418 | 263095 | 128804 | 84065 | 6257 | 40813 | 1918 |
|  |  | 2 | 324416 | 308391 | 264670 | 143621 | 94046 | 5601 | 45570 | 1862 |
|  |  | 3 | 293669 | 280470 | 238269 | 135727 | 85392 | 4182 | 46787 | 1733 |
|  |  | 4 | 297443 | 280295 | 226106 | 108485 | 70111 | 4005 | 35067 | 1455 |
|  |  | 5 | 366189 | 346508 | 293871 | 165774 | 107672 | 6130 | 54264 | 1918 |
|  |  | 6 | 377770 | 357022 | 292202 | 141418 | 92034 | 6166 | 45299 | 1831 |
| (+)infected/ (-)treated | 10  Spleen | 1 | 374662 | 361959 | 263884 | 180690 | 117619 | 6993 | 59638 | 2147 |
|  |  | 3 | 415000 | 393511 | 284581 | 135158 | 91225 | 7807 | 37889 | 2609 |

| Group | Day/ Organ | Mouse | %  Lymphocytes | Total # lymphoctyes/g | % CD3 | Total #CD3/g | % CD4 | Total # CD4/g |
| --- | --- | --- | --- | --- | --- | --- | --- | --- |
| (-)infected/ (+)treated | 10  Spleen | 1 | 85 | 619898553 | 53 | 329257565 | 64 | 212055090 |
|  |  | 2 | 82 | 681611732 | 64 | 436778549 | 65 | 284324692 |
|  |  | 3 | 84 | 660666634 | 58 | 382215062 | 64 | 246201208 |
|  |  | 4 | 79 | 638718061 | 57 | 366721410 | 65 | 239131870 |
|  |  | 5 | 80 | 320961152 | 57 | 183074657 | 66 | 120069332 |
|  |  | 6 | 79 | 593675137 | 54 | 323154770 | 64 | 208113927 |
| (+)infected/ (+)treated | 10  Spleen | 1 | 83 | 673134574 | 49 | 329547980 | 65 | 215082225 |
|  |  | 2 | 86 | 695564405 | 54 | 377442307 | 65 | 247157026 |
|  |  | 3 | 85 | 603908370 | 57 | 344008962 | 63 | 216431611 |
|  |  | 4 | 81 | 691919675 | 48 | 331981044 | 65 | 214550611 |
|  |  | 5 | 85 | 595508762 | 56 | 335929266 | 65 | 218189680 |
|  |  | 6 | 82 | 602852813 | 48 | 291764735 | 65 | 189878768 |
| (+)infected/ (-)treated | 10  Spleen | 1 | 73 | 368896212 | 68 | 252595294 | 65 | 164425291 |
|  |  | 3 | 72 | 321692352 | 47 | 152783548 | 67 | 103121378 |

| Group | Day/ Organ | Mouse | % CD4  CD69 | Total # CD4CD69/g | % CD8 | Total # CD8/g | % CD8CD69 | Total # CD8CD69/g |
| --- | --- | --- | --- | --- | --- | --- | --- | --- |
| (-)infected/ (+)treated | 10  Spleen | 1 | 4 | 8282413 | 33 | 108456022 | 3 | 3630762 |
|  |  | 2 | 4 | 11923161 | 33 | 142212694 | 4 | 4993779 |
|  |  | 3 | 4 | 9800203 | 33 | 127359749 | 3 | 4188854 |
|  |  | 4 | 4 | 10399872 | 32 | 118422245 | 3 | 3999951 |
|  |  | 5 | 4 | 5319248 | 32 | 58389579 | 4 | 2338976 |
|  |  | 6 | 4 | 8523818 | 33 | 107224122 | 4 | 3849246 |
| (+)infected/ (+)treated | 10  Spleen | 1 | 7 | 16008678 | 32 | 104420994 | 5 | 4907247 |
|  |  | 2 | 6 | 14719674 | 32 | 119759965 | 4 | 4893418 |
|  |  | 3 | 5 | 10599553 | 34 | 118584713 | 4 | 4392402 |
|  |  | 4 | 6 | 12255926 | 32 | 107310497 | 4 | 4452527 |
|  |  | 5 | 6 | 12422011 | 33 | 109962152 | 4 | 3886691 |
|  |  | 6 | 7 | 12721304 | 32 | 93458052 | 4 | 3777604 |
| (+)infected/ (-)treated | 10  Spleen | 1 | 6 | 9775853 | 33 | 83370846 | 4 | 3001395 |
|  |  | 3 | 9 | 8825087 | 28 | 42829991 | 7 | 2949232 |
